# Supplementary material for: Degradation of the TEAD•YAP/TAZ Transcription Factor Complex by Heterobifunctional Small Molecules that Bind to the TEAD Allosteric Lipid Pocket
Source: bioRxiv. 2026 Jan 11:2026.01.09.698724. Preprint. [Version 1] doi: 10.64898/2026.01.09.698724 (PMC12803072; doi:10.64898/2026.01.09.698724)
Supplement: Supplement 1 [file media-1.pdf]

Degradation of the TEAD•YAP/TAZ Transcription Factor Complex by Heterobifunctional Small Molecules that Bind to the TEAD Allosteric Lipid Pocket

I-Ju Yeh[3], Mona K. Ghozayel[1], Khuchtumur Bum-Erdene[3], and Samy O. Meroueh[1,2,3]*

[1] Department of Biochemistry, University of Illinois Urbana-Champaign, Urbana, IL, 61801, USA

[2] Cancer Center at Illinois, University of Illinois Urbana-Champaign, Urbana, IL, 61801, USA

[3] Department of Veterans Affairs, Richard L. Roudebush VA Medical Center, Indianapolis, Indiana, 46202

*Corresponding author

Corresponding Author and Lead Contact:

Samy Meroueh

Department of Biochemistry

University of Illinois Urbana Champaign

600 S. Matthews Ave.

Urbana, IL 61801

E-mail: smeroueh@illinois.edu

^{1}H NMR (400 MHz) of compound TED-650

TED-549-4

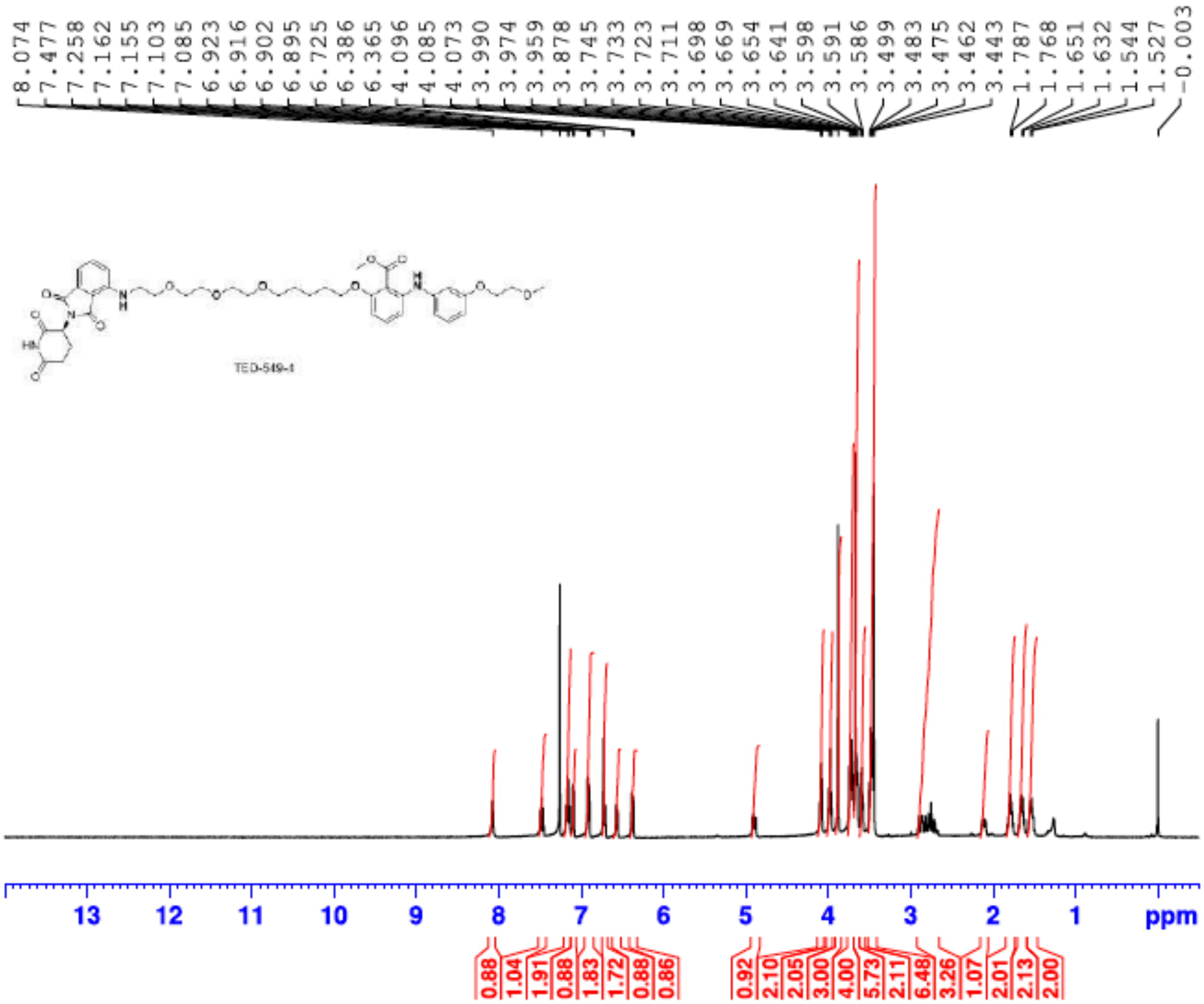

^{1}H NMR (400 MHz) of compound TED-651

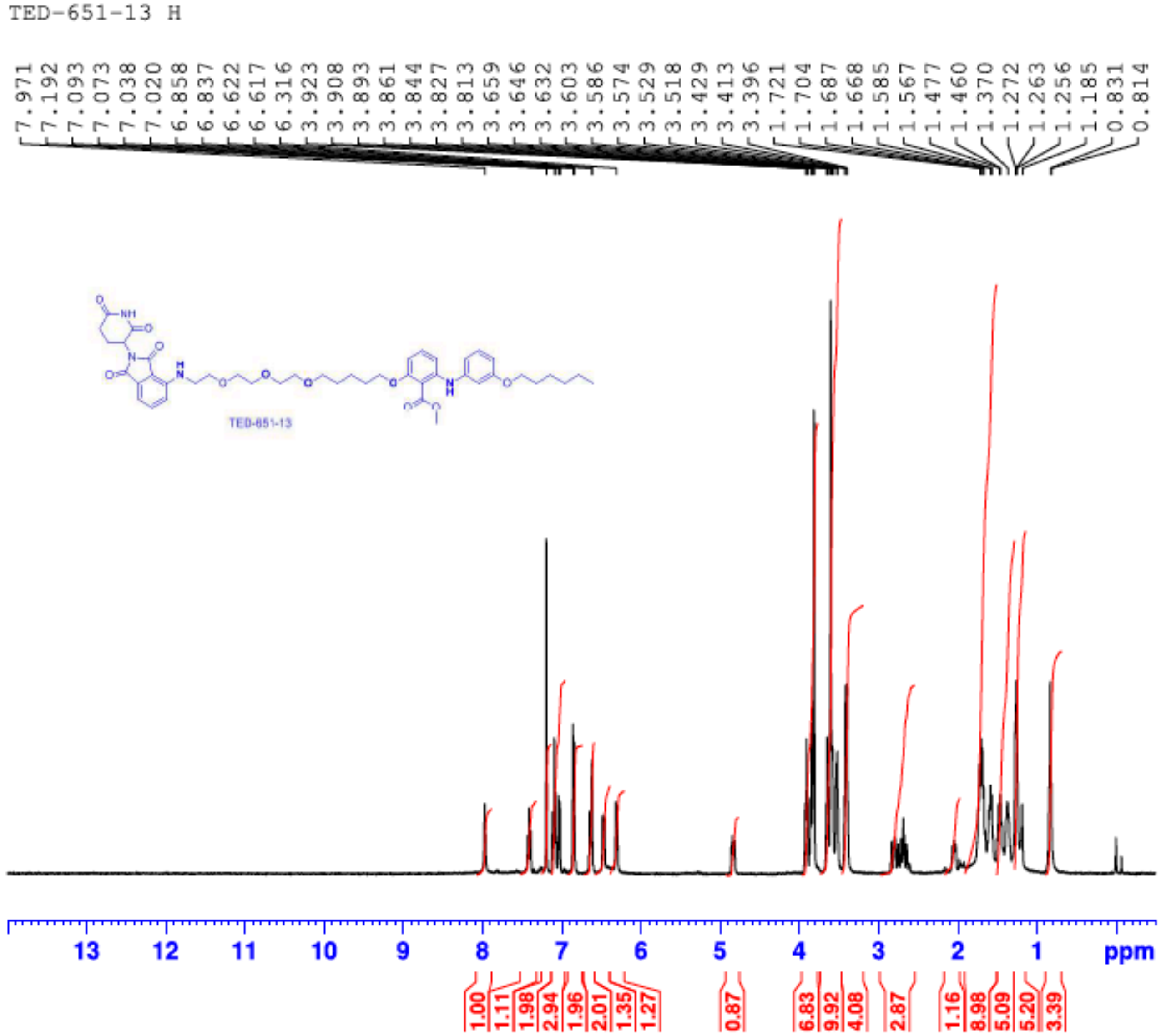

^{13}C NMR (100 MHz) of compound TED-651

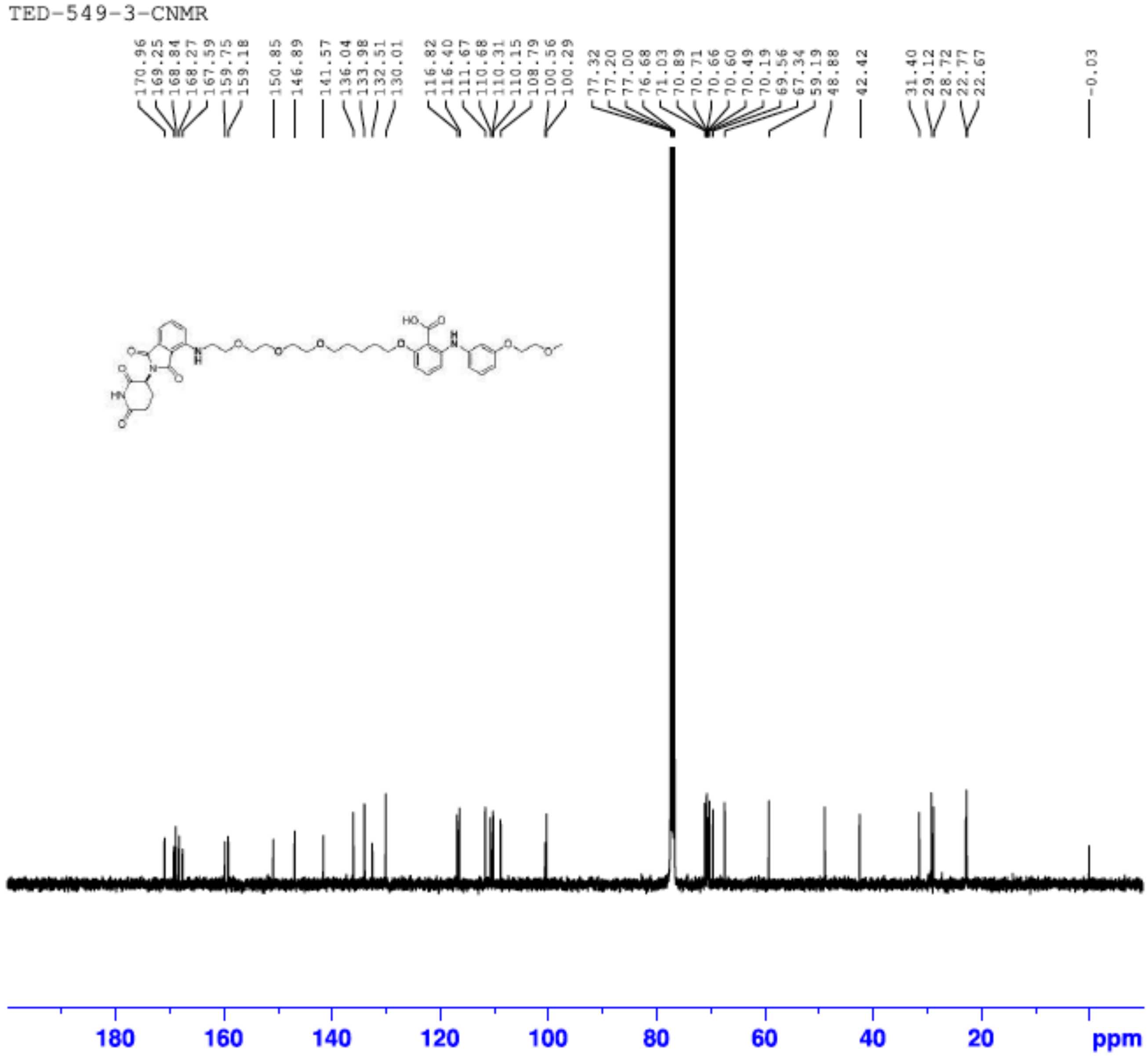

^{1}H NMR (400 MHz) of compound TED-652

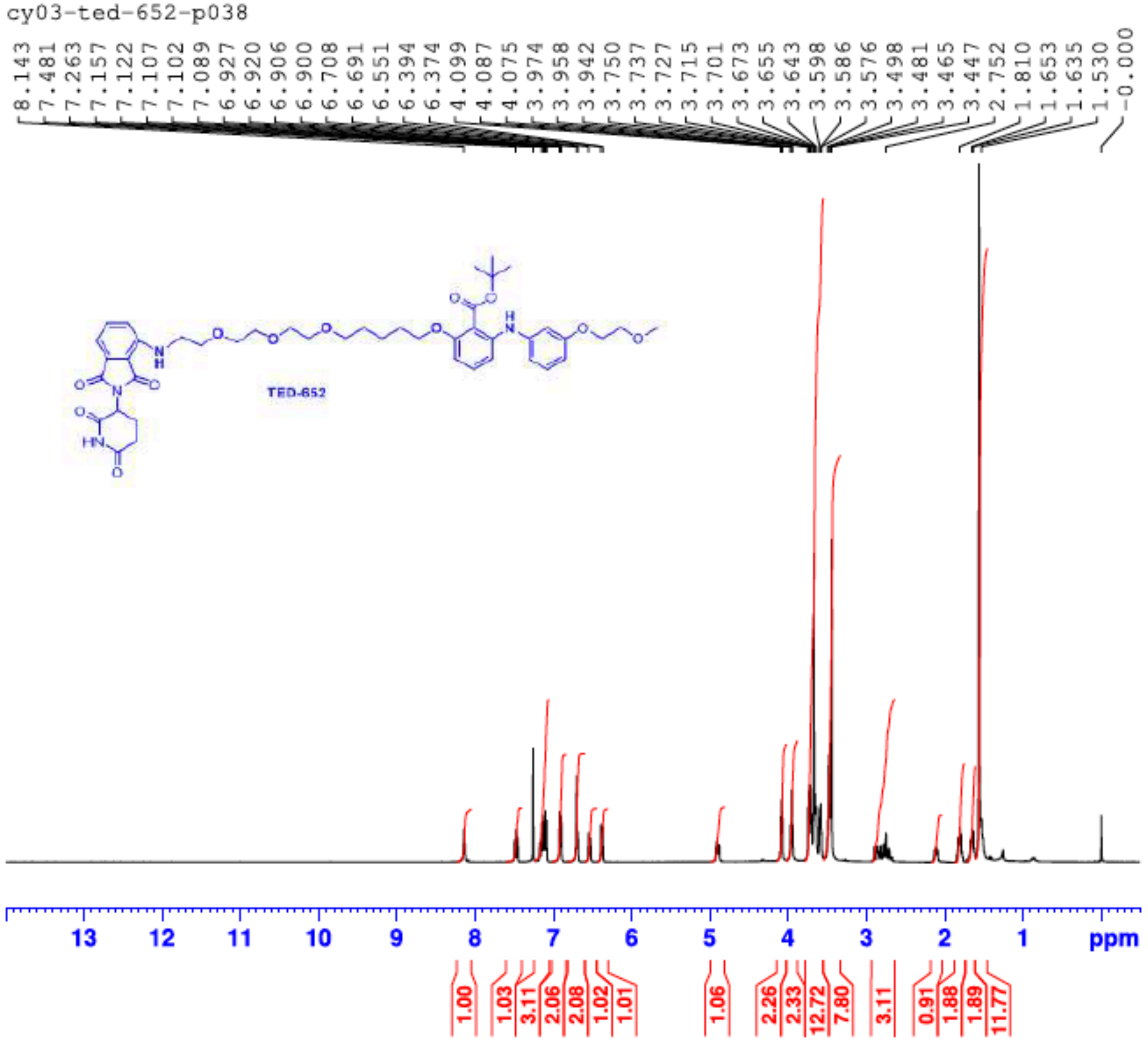

^{13}C NMR (100 MHz) of compound TED-652

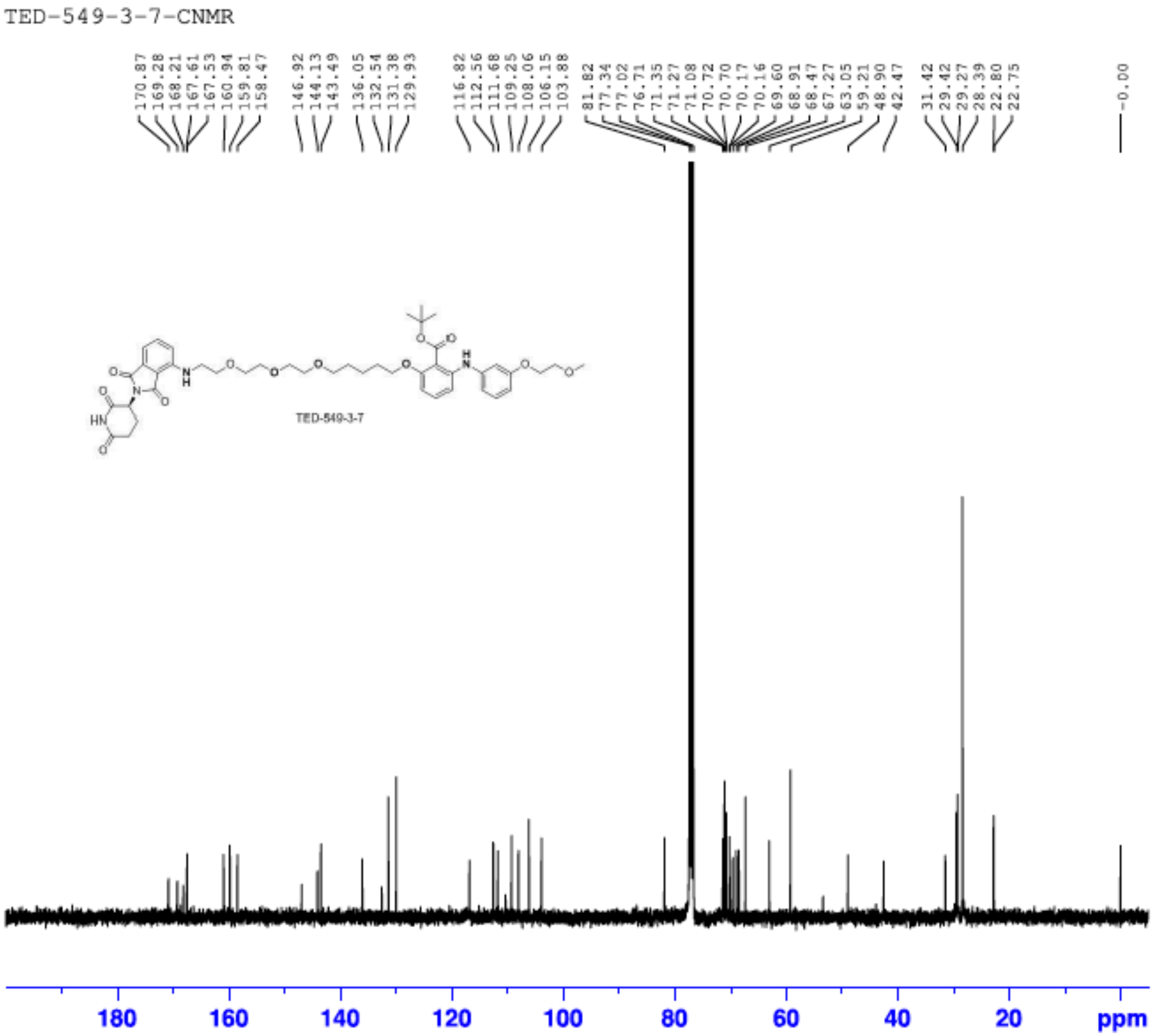

^{1}H NMR (400 MHz) of compound TED-670

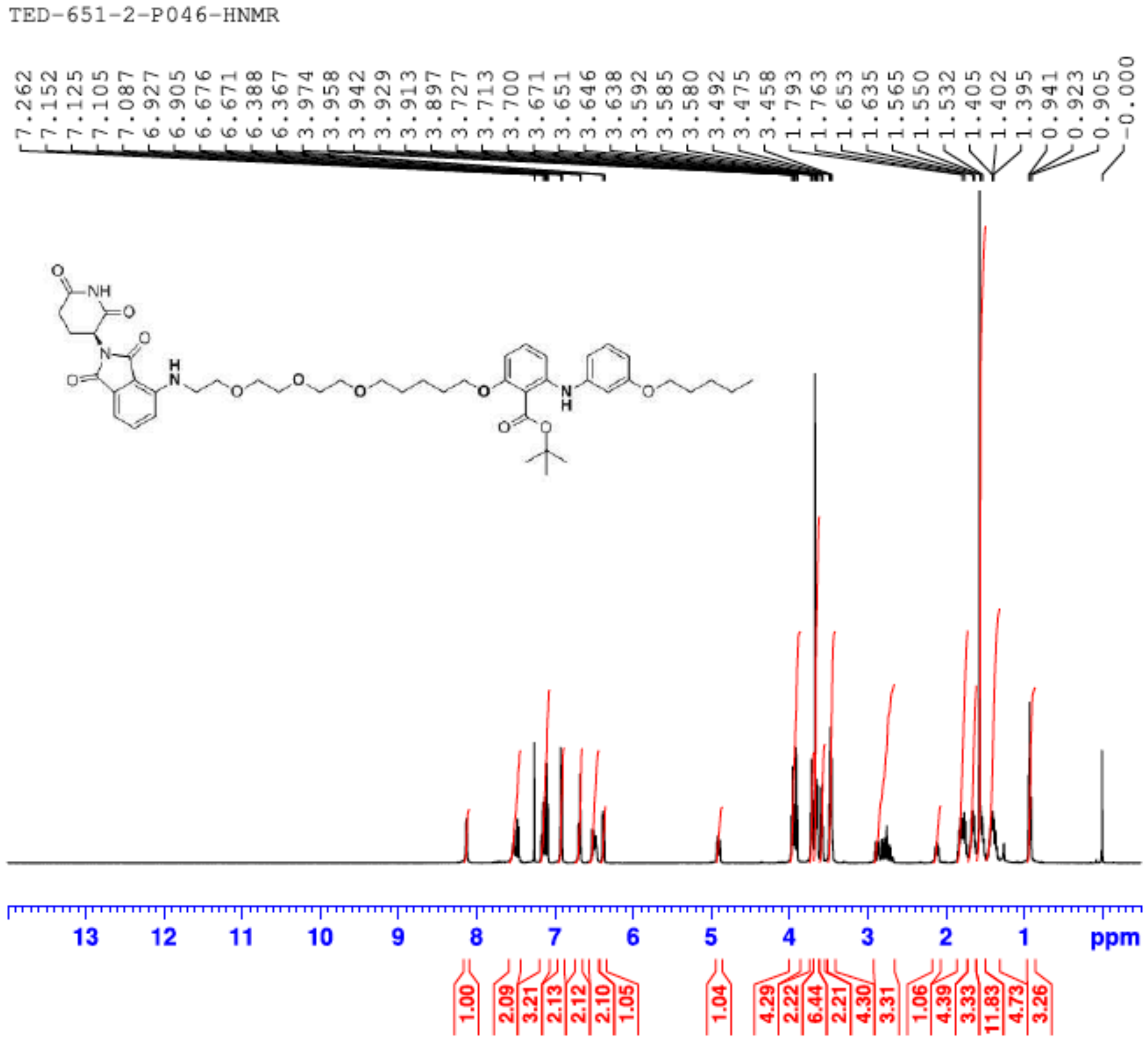

^{13}C NMR (100 MHz) of compound TED-670

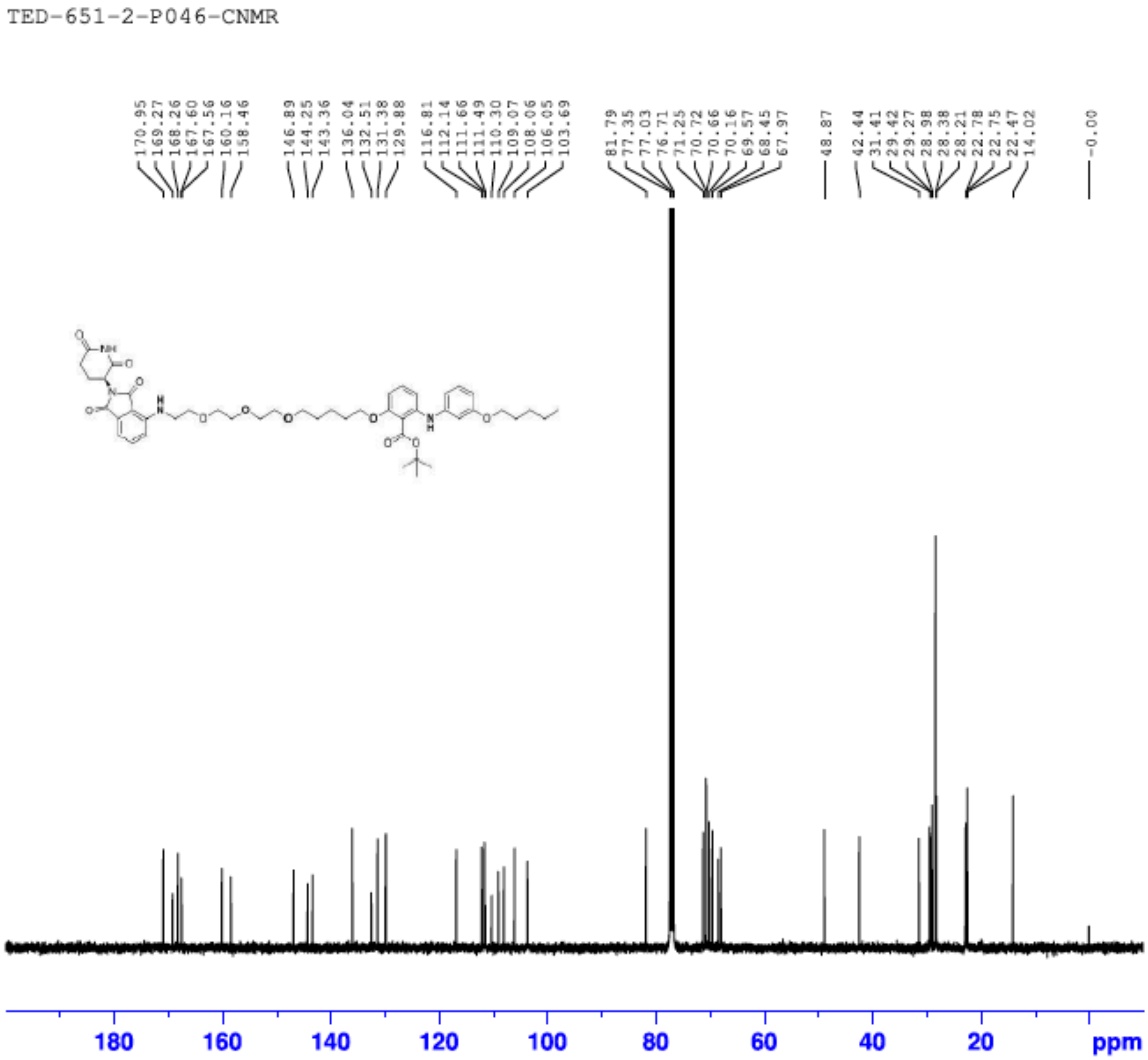

^{1}H NMR (400 MHz) of compound TED-671

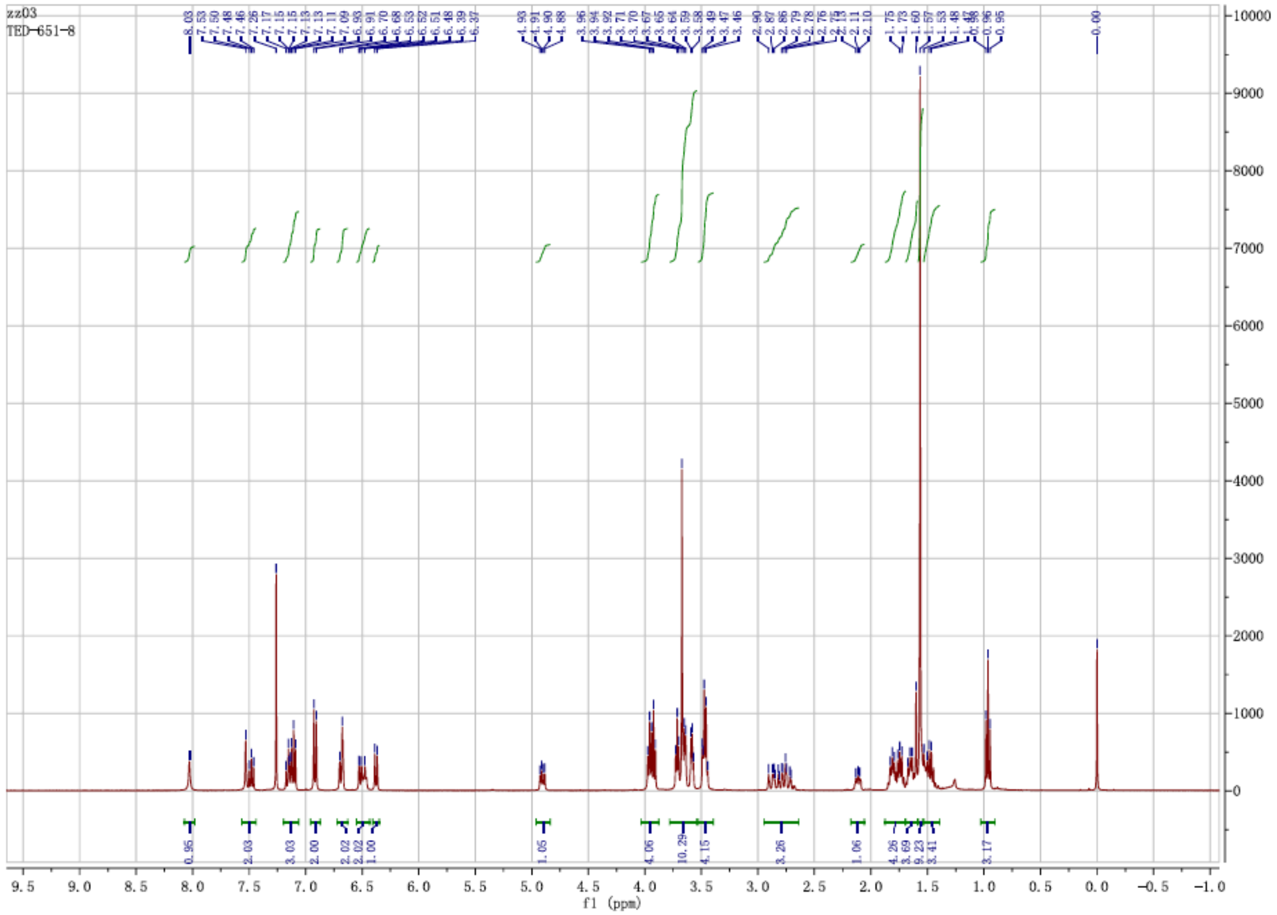

^{13}C NMR (100 MHz) of compound TED-671

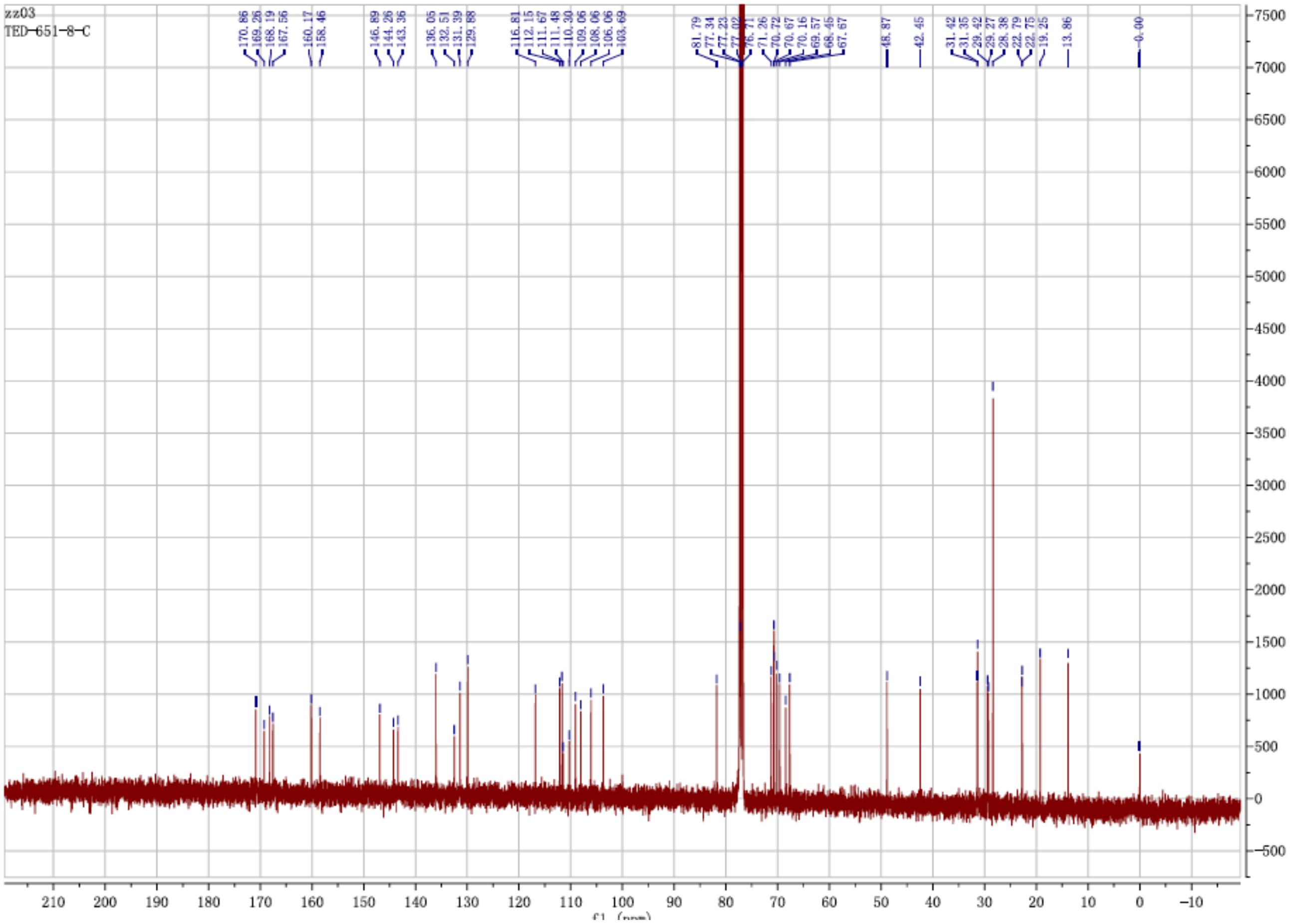

^{1}H NMR (400 MHz) of compound TED-672

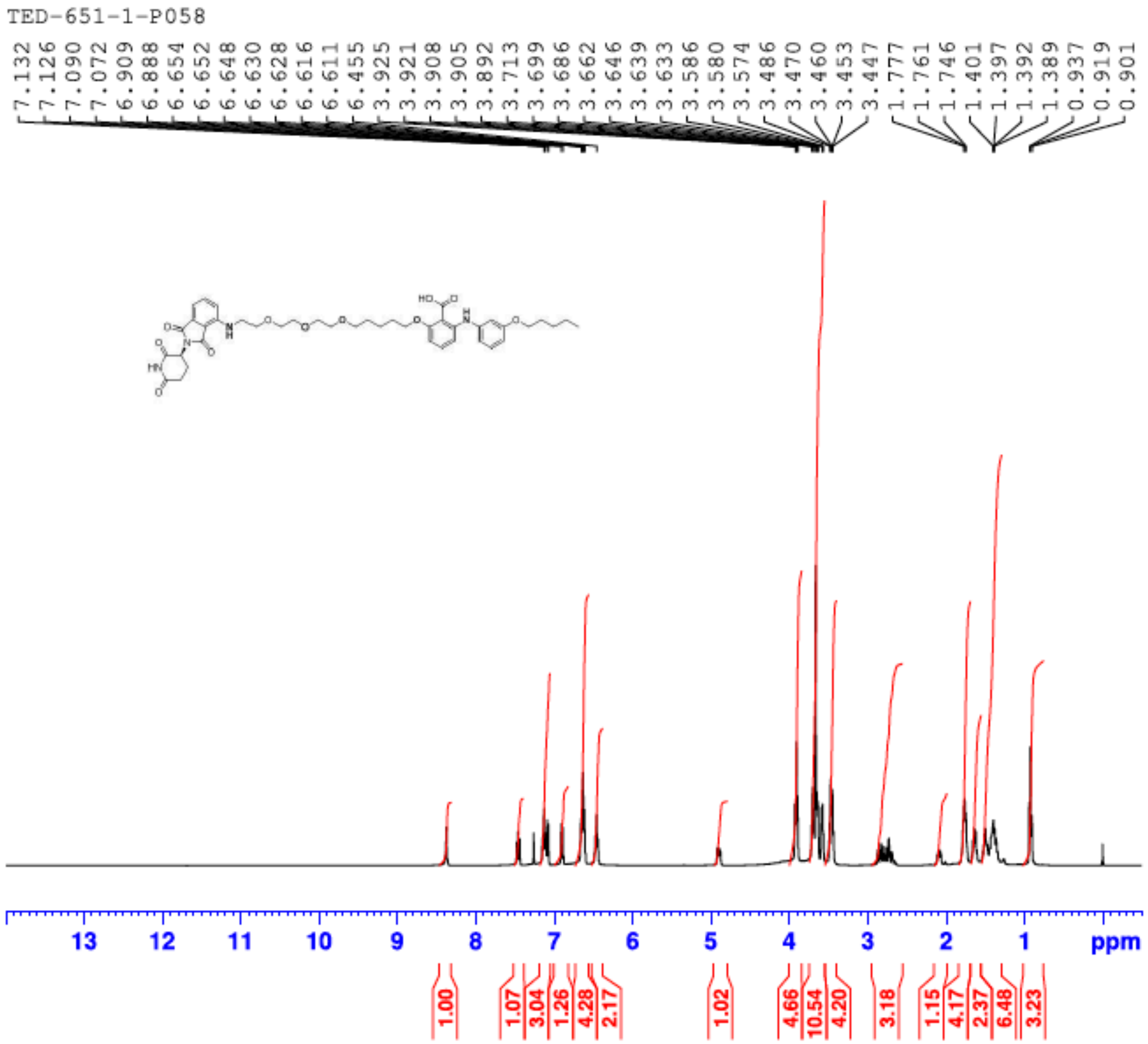

^{13}C NMR (100 MHz) of compound TED-672

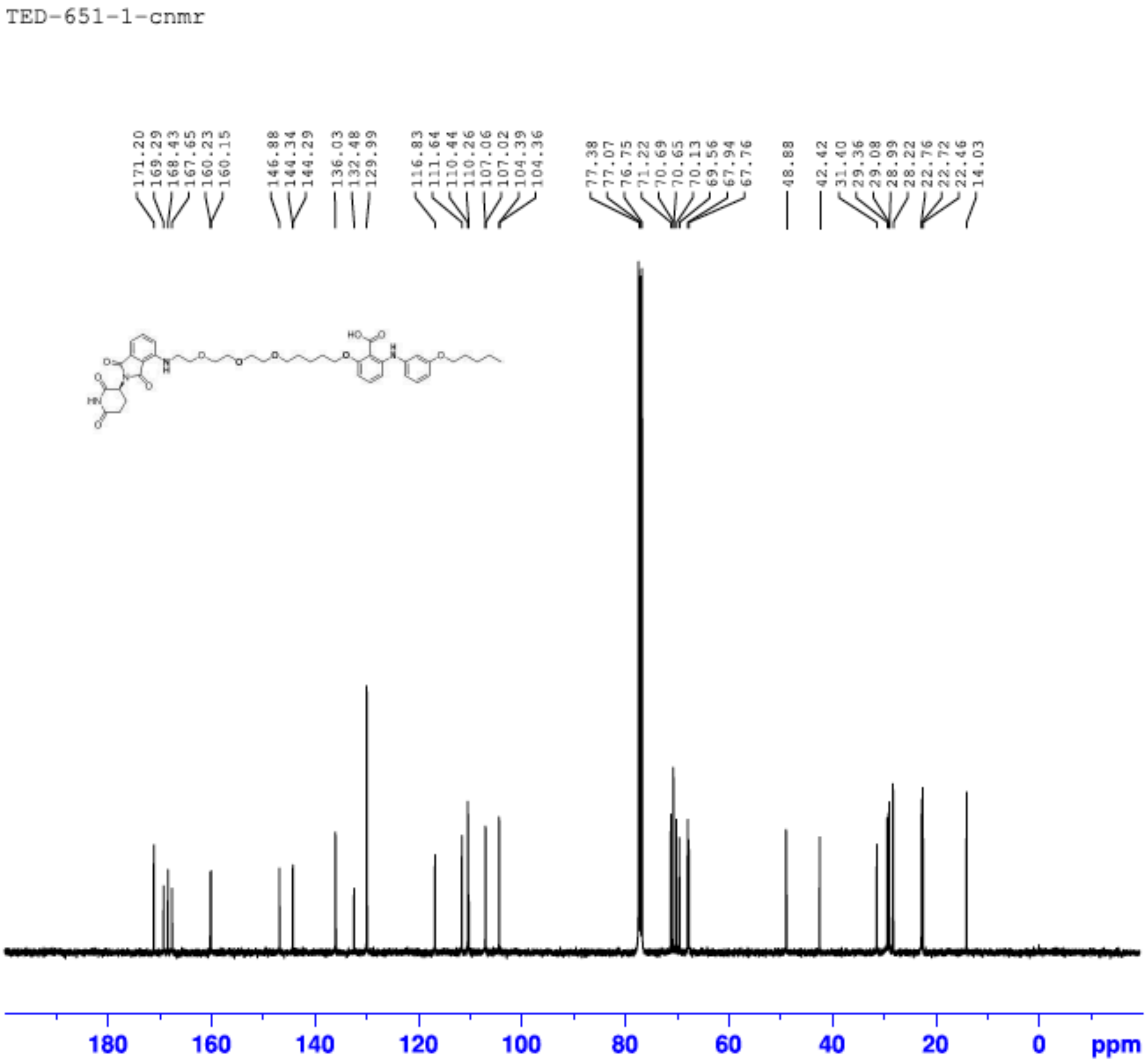

^{1}H NMR (400 MHz) of compound TED-673

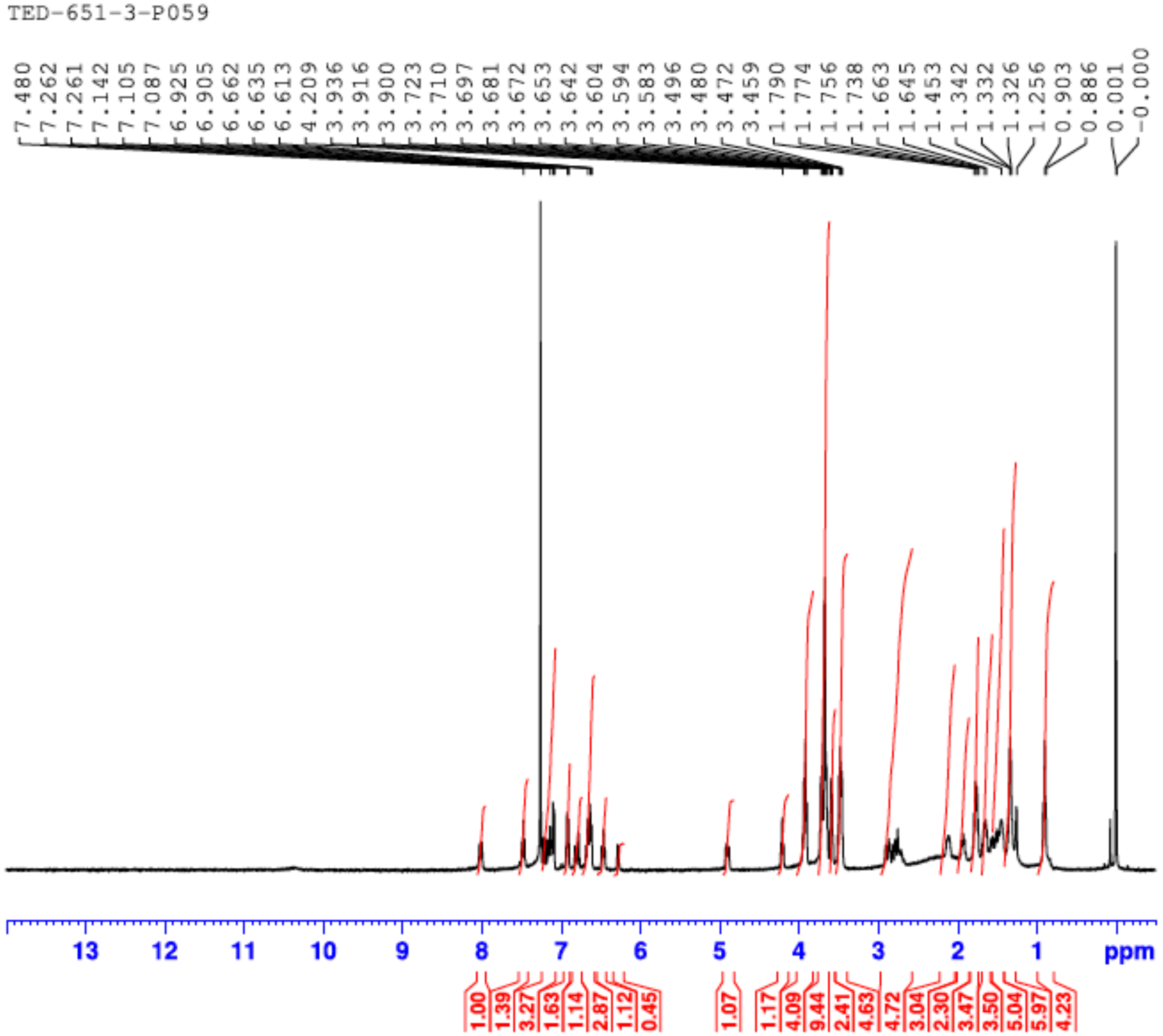

^{1}H NMR (400 MHz) of compound TED-674

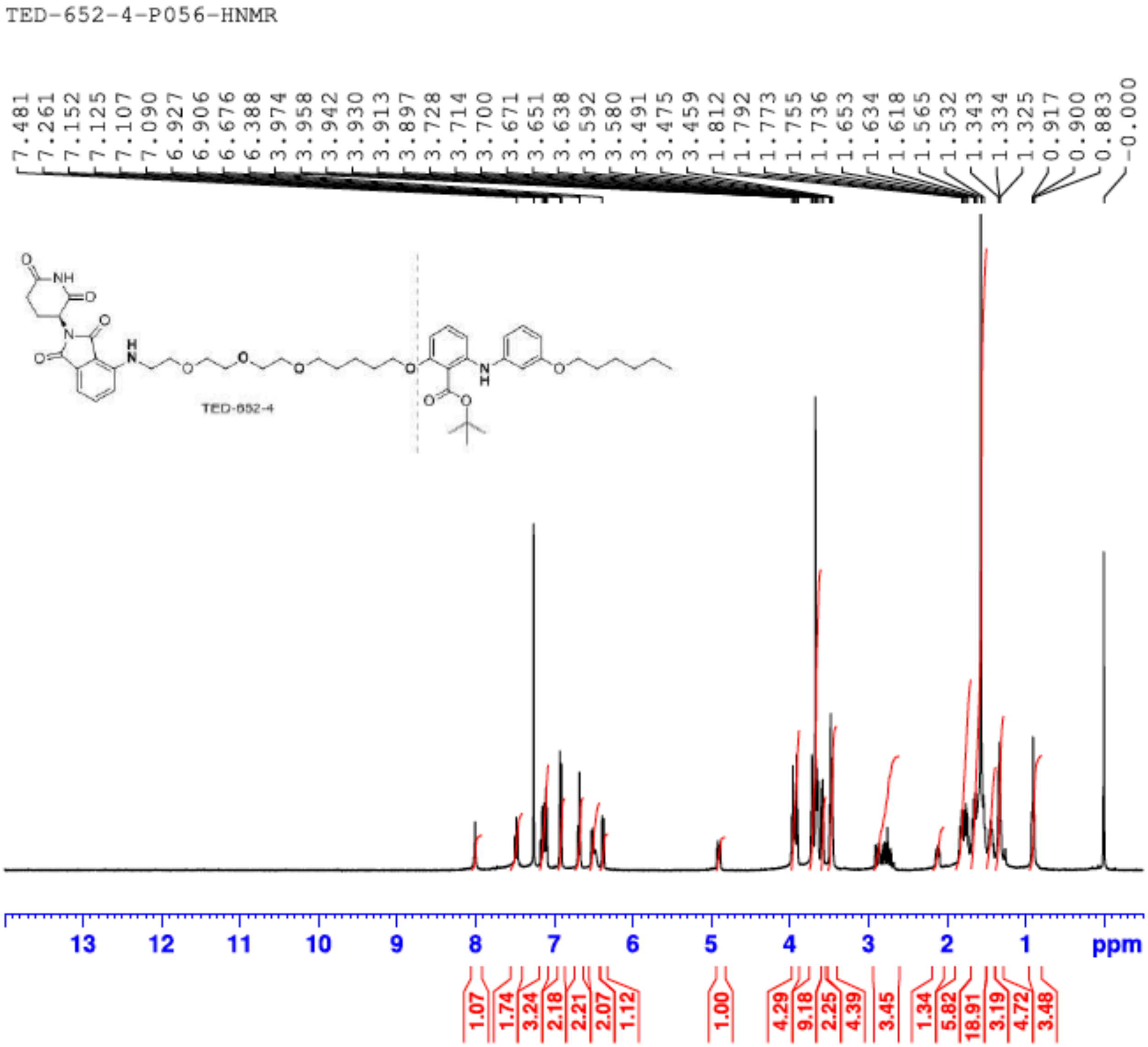

^{1}H NMR (400 MHz) of compound TED-675

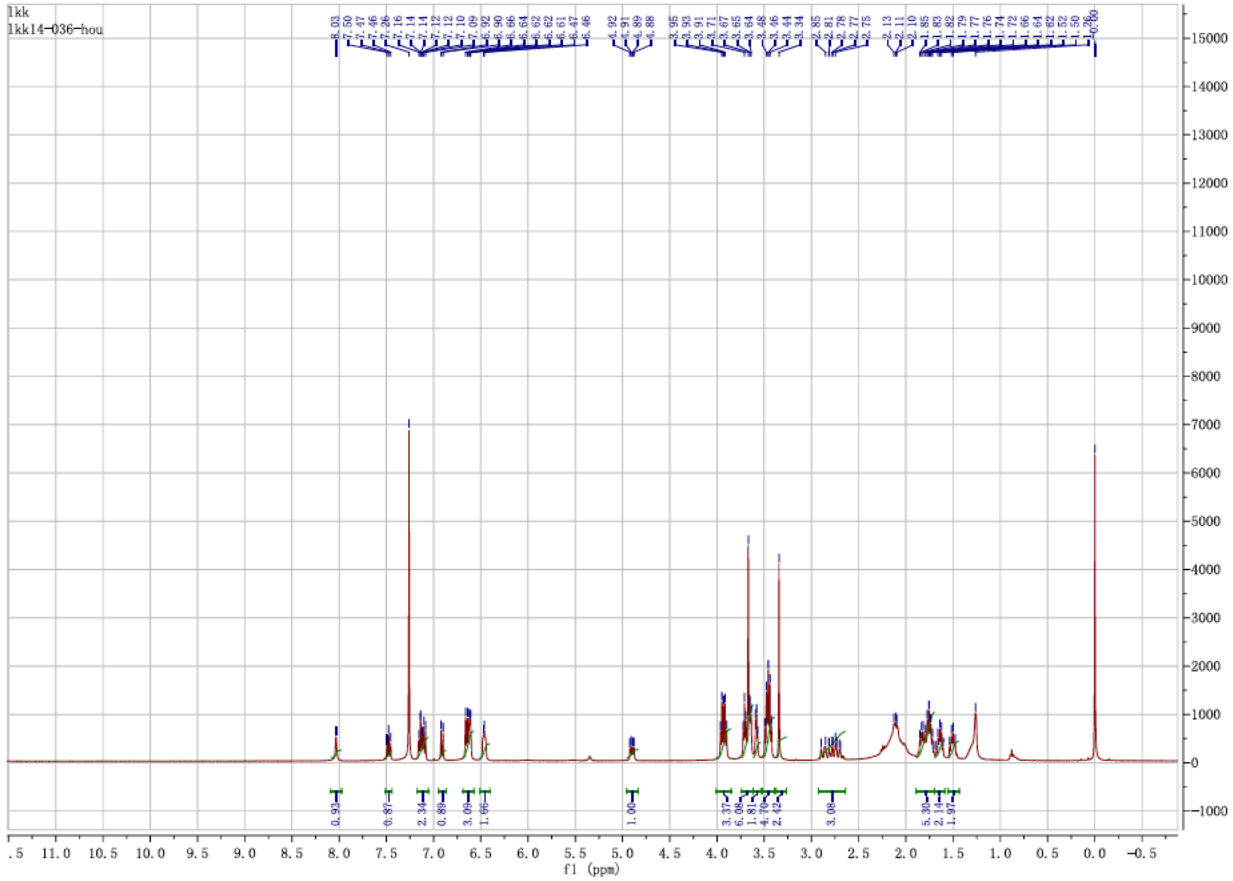

^{1}H NMR (400 MHz) of compound TED-676

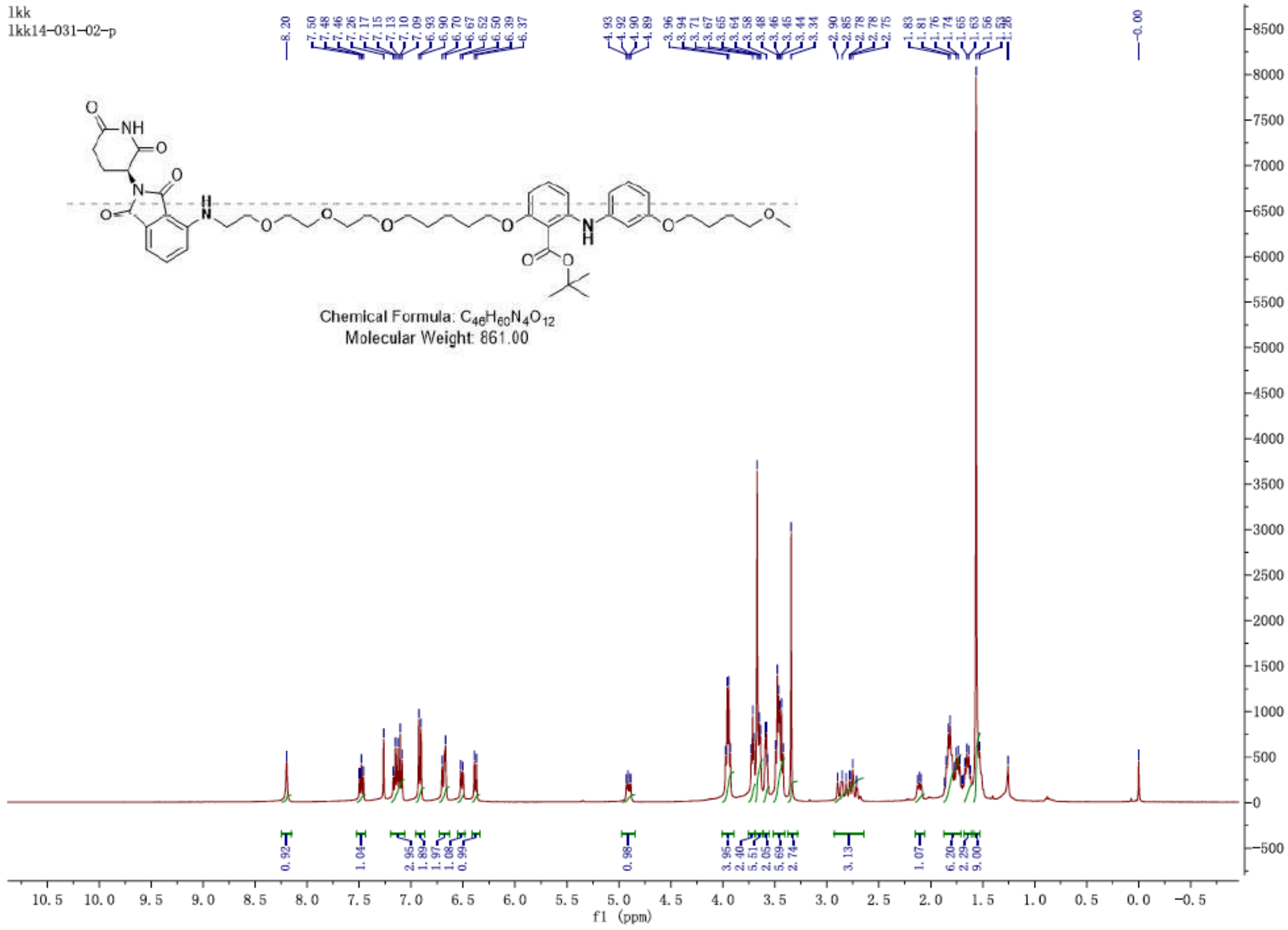

^{1}H NMR (400 MHz) of compound TED-677

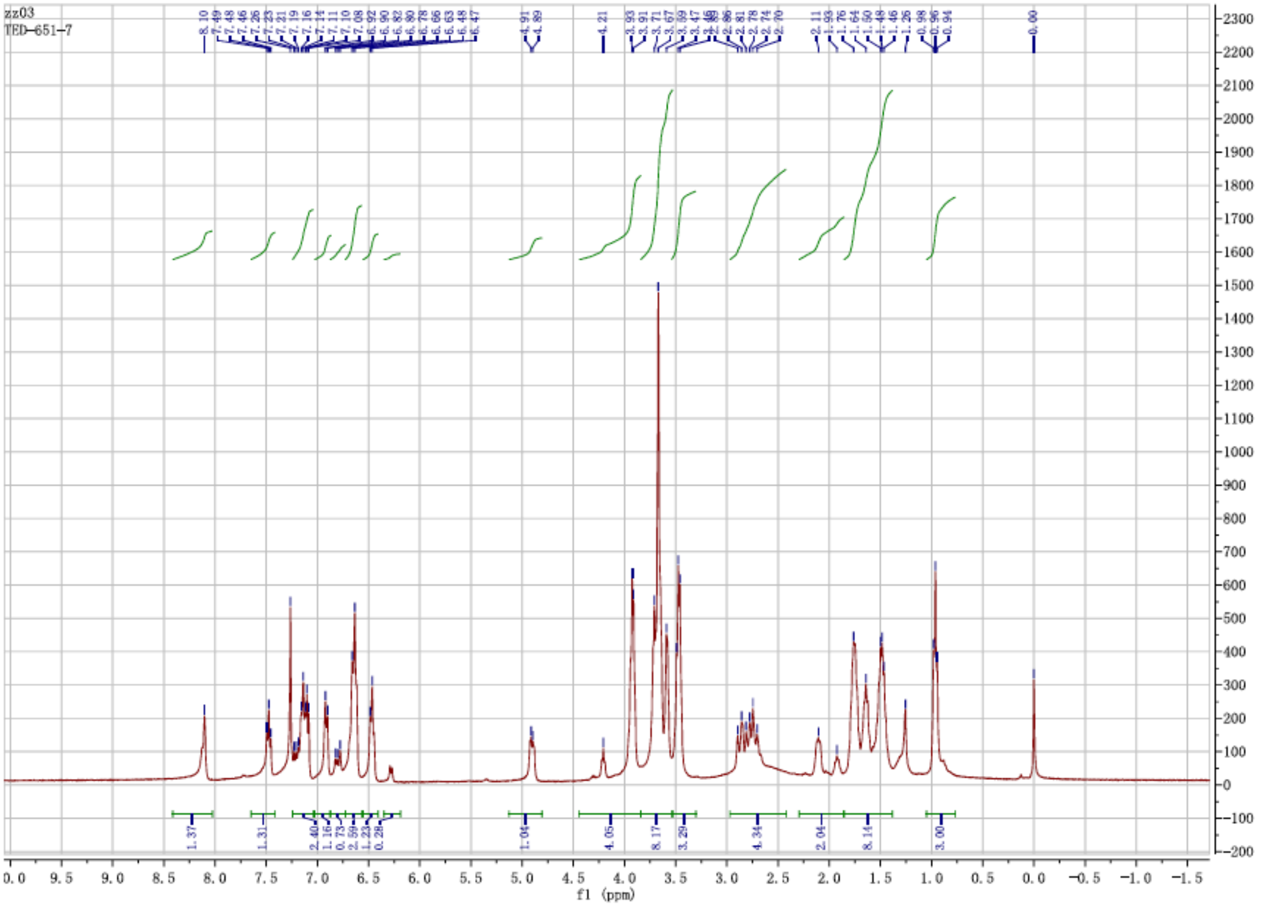

^{1}H NMR (400 MHz) of compound TED-688

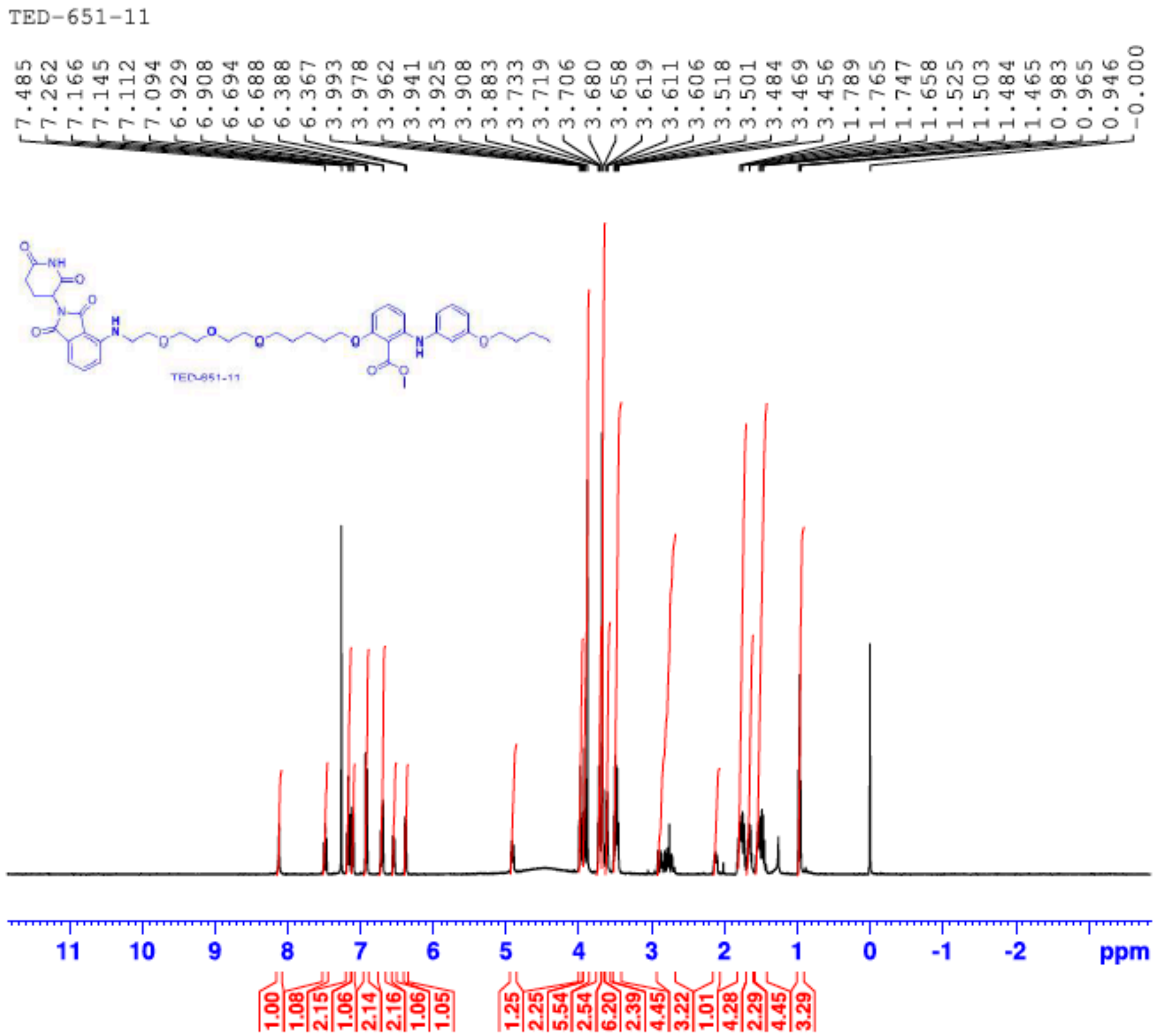

^{1}H NMR (400 MHz) of compound TED-689

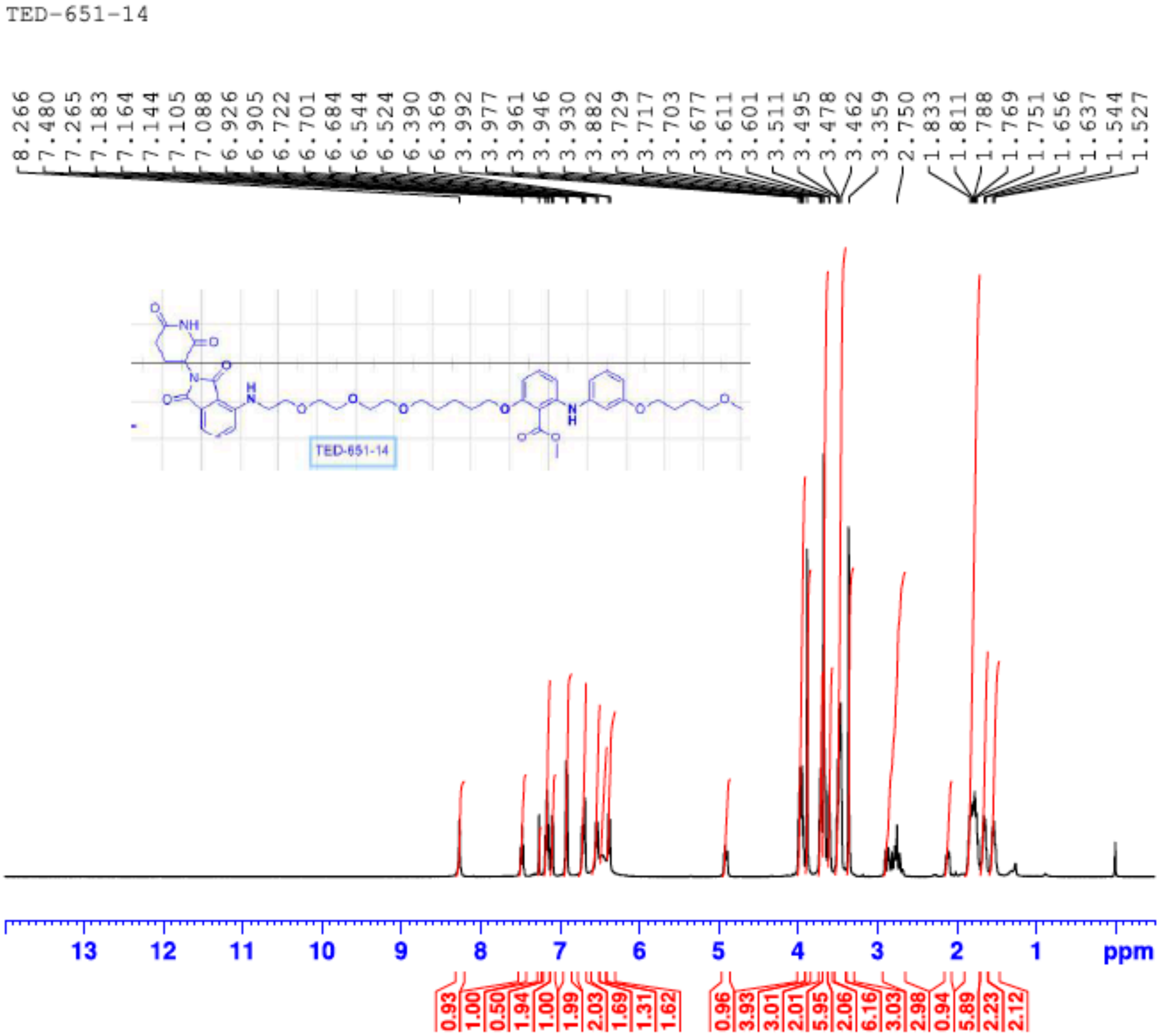

^{13}C NMR (100 MHz) of compound TED-689

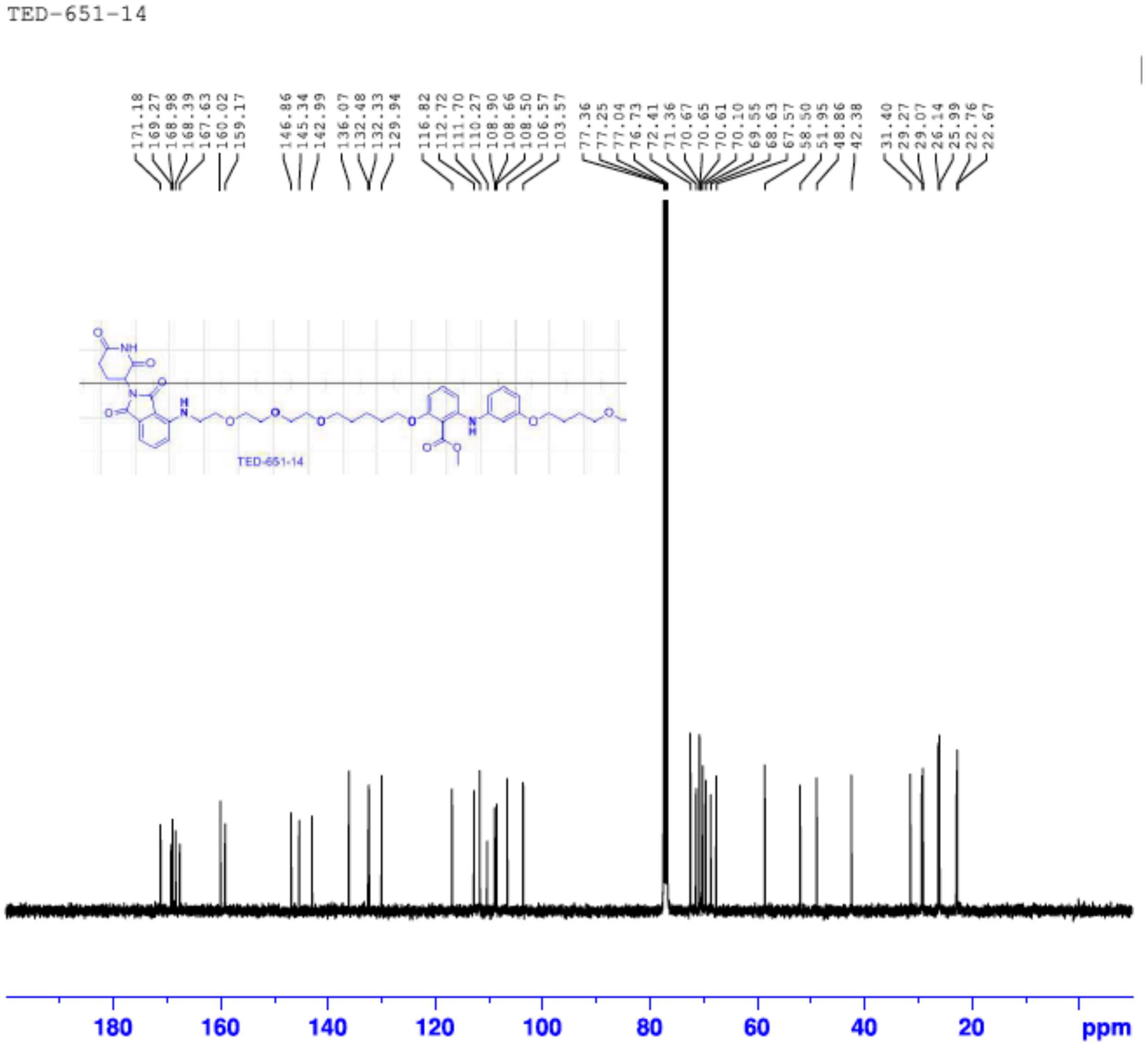

^{1}H NMR (400 MHz) of compound TED-690

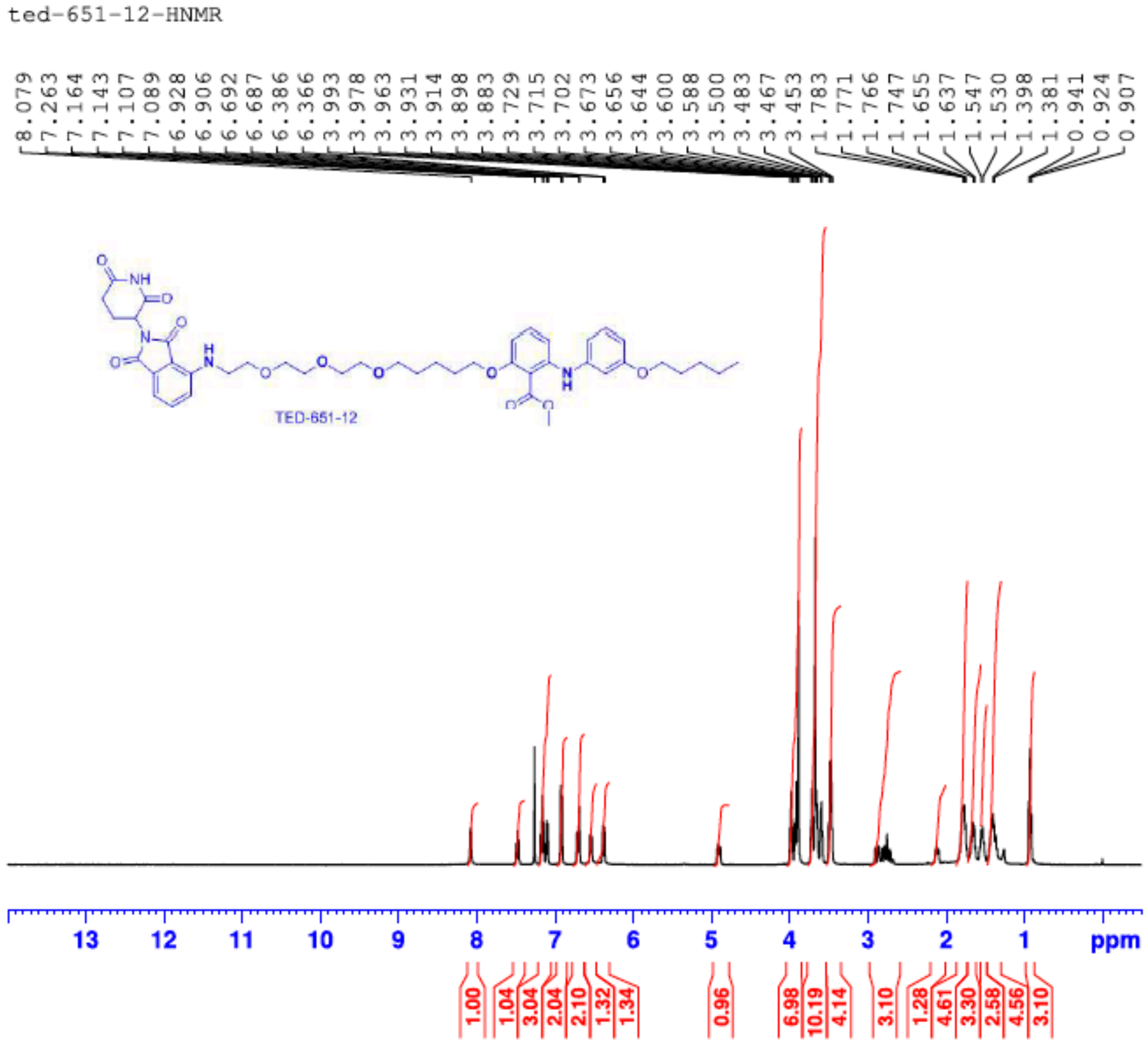

^{13}C NMR (100 MHz) of compound TED-690

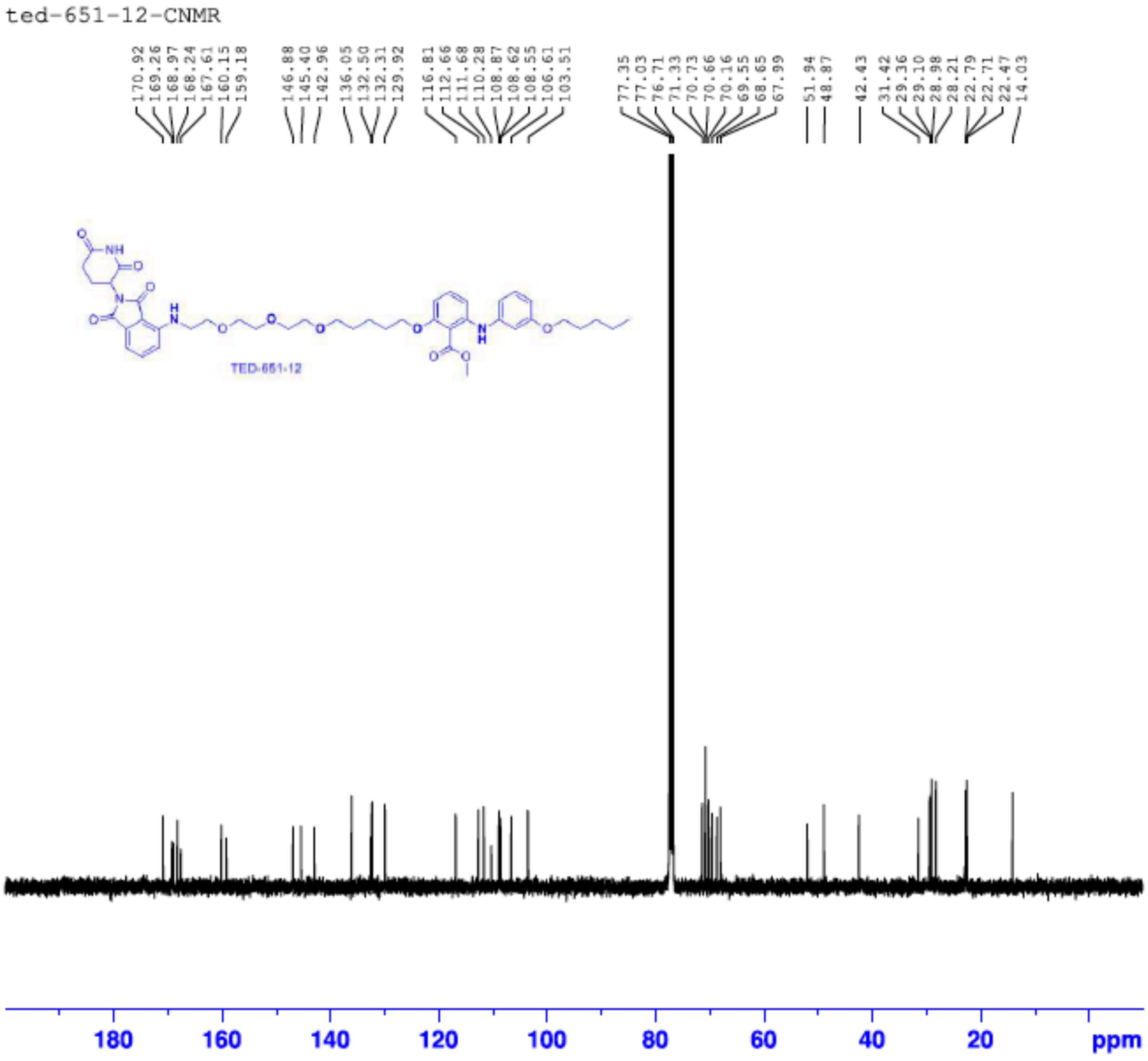

^{1}H NMR (400 MHz) of compound TED-734

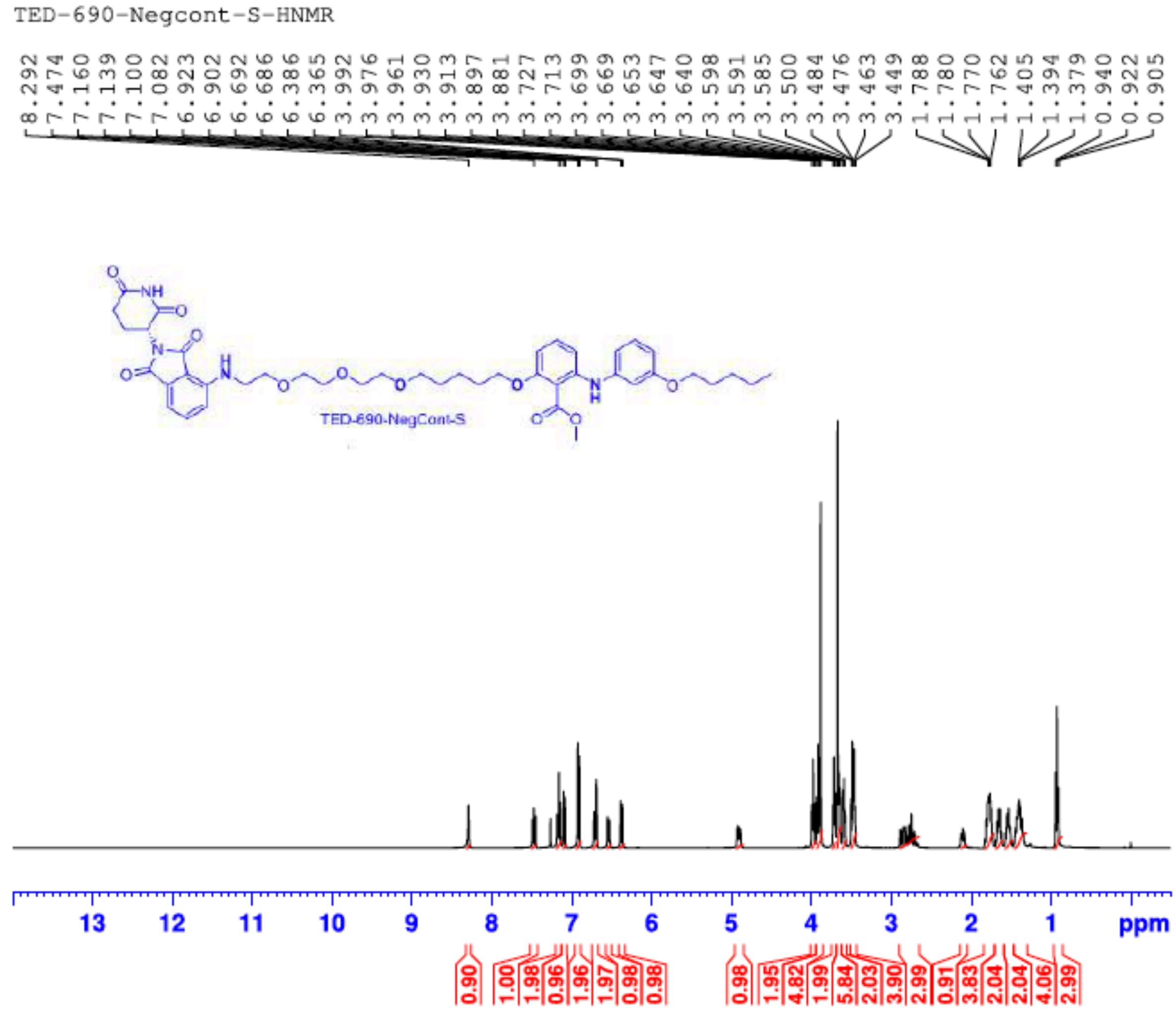

^{13}C NMR (100 MHz) of compound TED-734

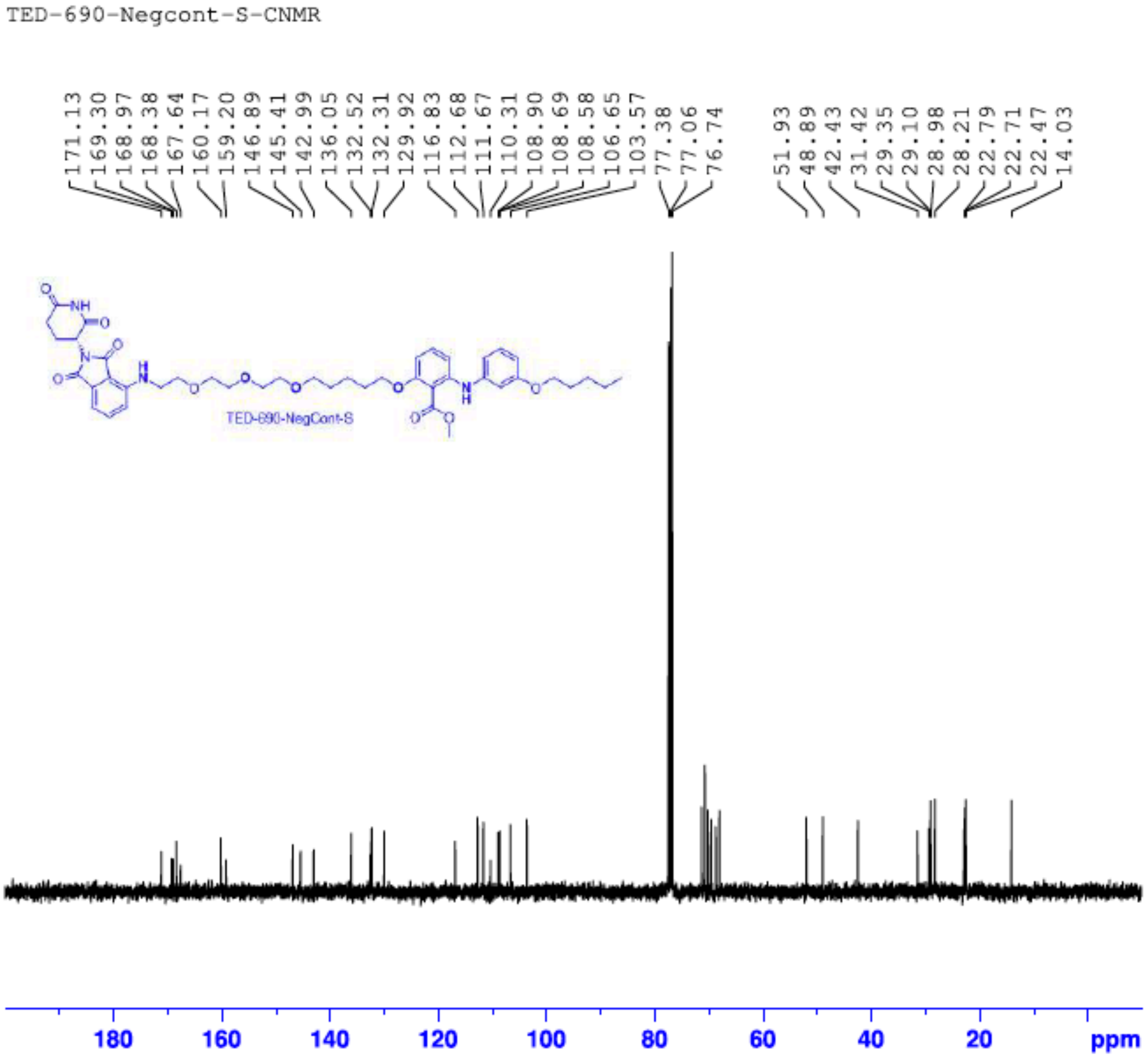

HPLC Traces:

HPLC TED-650

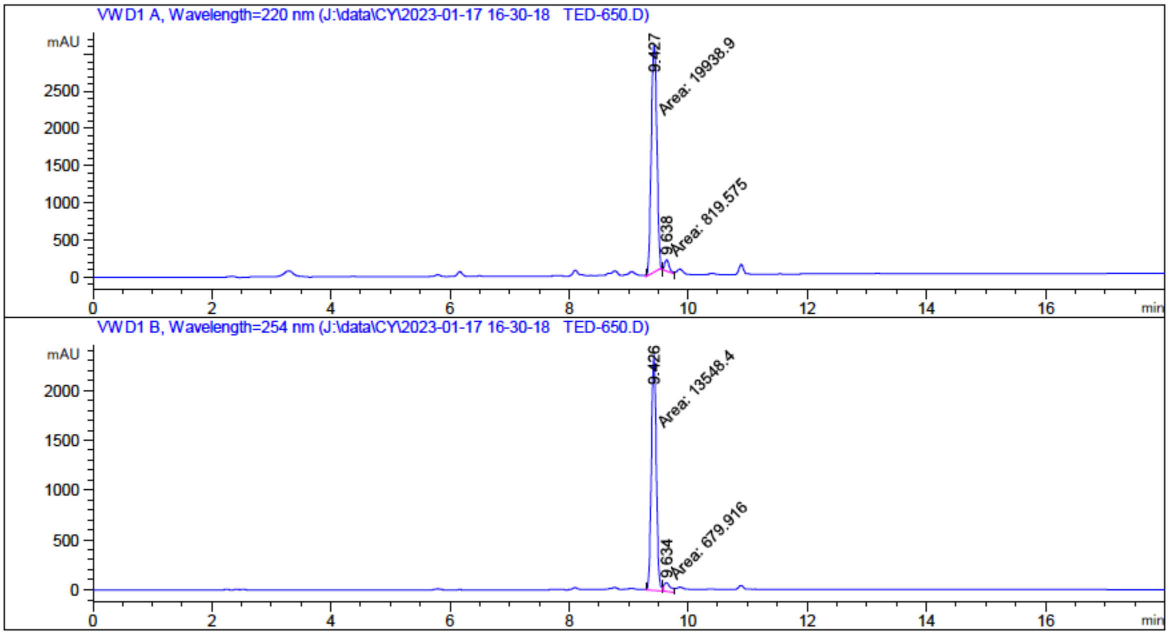

UV 220 nm:

RT: 9.427 min **Area %: 96.0518 %**

RT: 9.638 min **Area %: 3.9482 %**

UV 254 nm:

RT: 9.426 min **Area %: 95.2214 %**

RT: 9.634 min **Area %: 4.7786 %**

HPLC TED-651

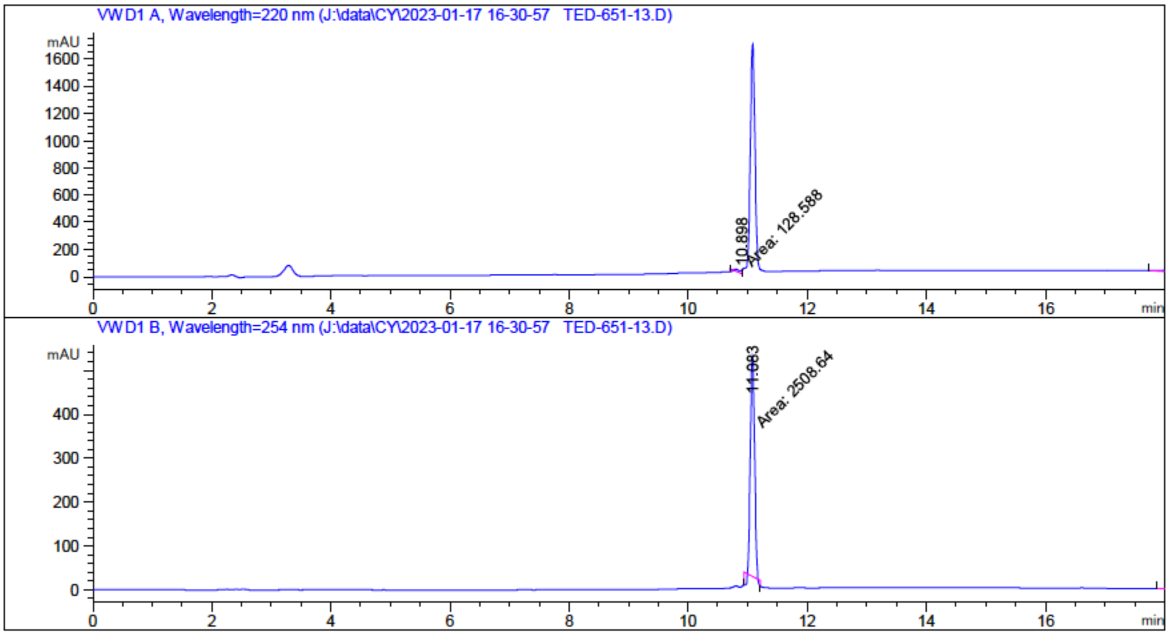

UV 220 nm:

RT: 10.898 min **Area %: 6.6483 %**

RT: 18.130 min **Area %: 93.3517 %**

UV 254 nm:

RT: 11.083 min **Area %: 96.1843 %**

RT: 18.129 min **Area %: 3.8157 %**

HPLC TED-652

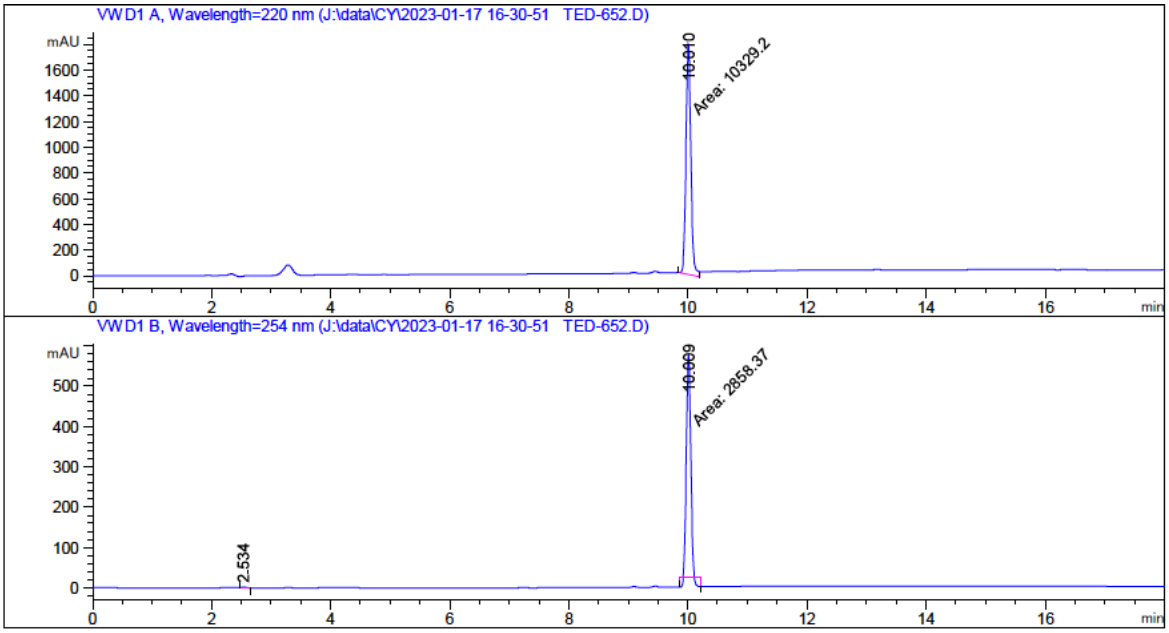

UV 220 nm:

RT: 10.010 min **Area %: 100.0000 %**

UV 254 nm:

RT: 2.534 min **Area %: 0.3371 %**

RT: 10.009 min **Area %: 99.6629 %**

HPLC TED-670

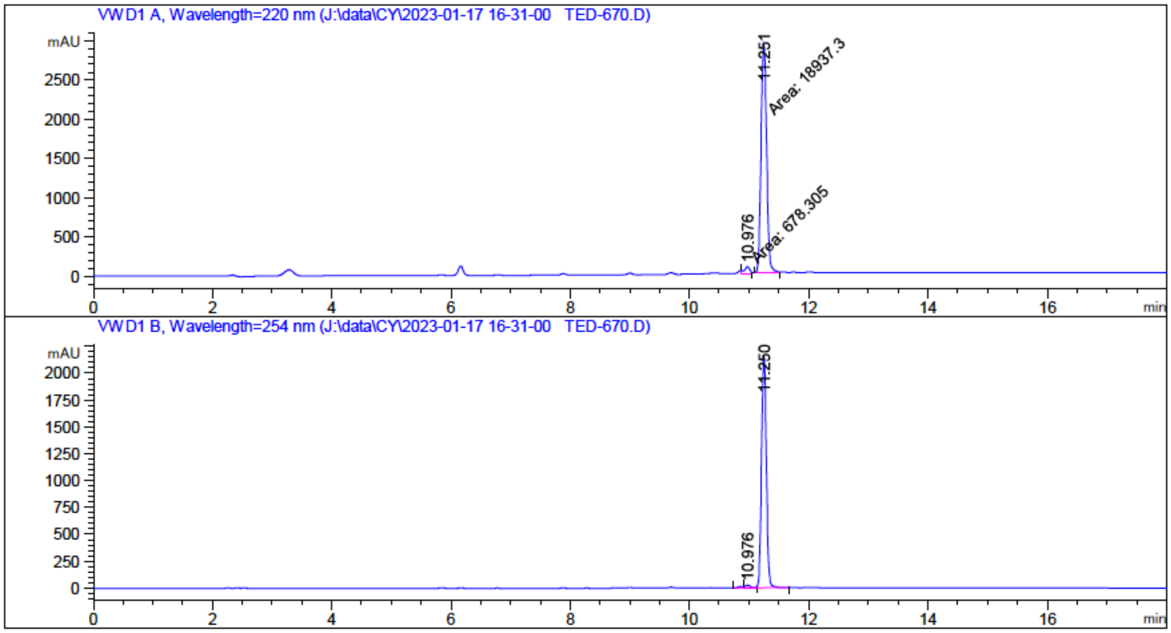

UV 220 nm:

RT: 10.976 min Area %: 3.4580 %

RT: 11.251 min Area %: 96.5420 %

UV 254 nm:

RT: 10.976 min Area %: 1.2236 %

RT: 11.250 min Area %: 98.7764 %

HPLC TED-671

UV 220 nm:

RT: min Area %: %

UV 254 nm:

RT: min Area %: %

HPLC TED-672

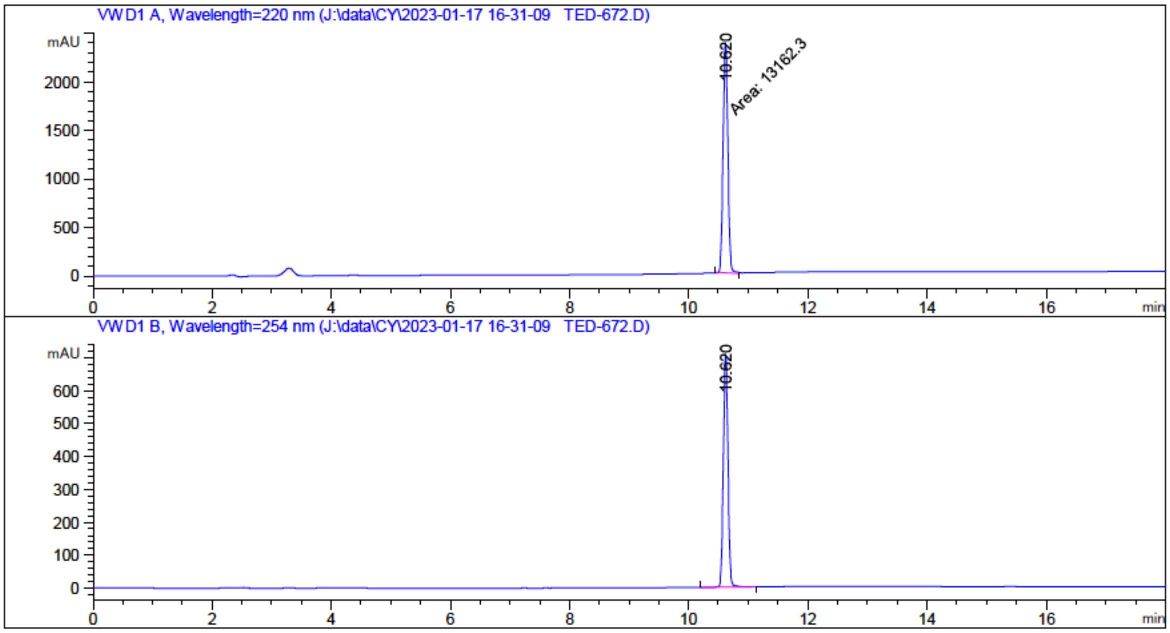

UV 220 nm:

RT: 10.620 min Area %: 100.0000 %

UV 254 nm:

RT: 10.620 min Area %: 100.0000 %

HPLC TED-673

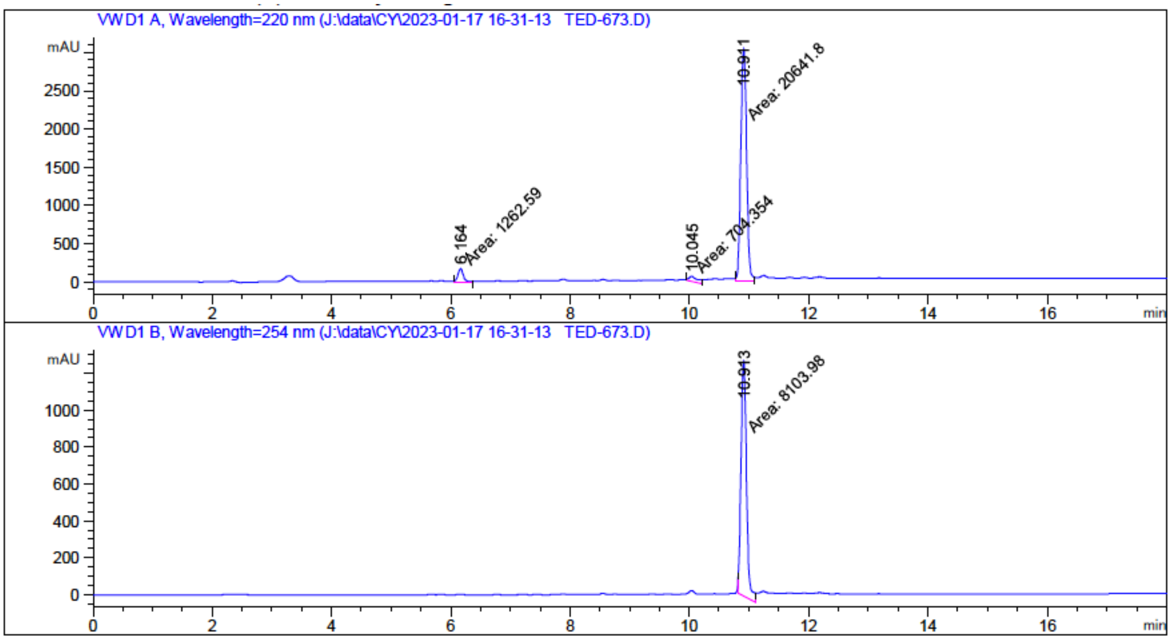

UV 220 nm:

RT: 6.164 min **Area %: 5.5845 %**

RT: 10.045 min **Area %: 3.1154 %**

RT: 10.911 min **Area %: 91.3001 %**

UV 254 nm:

RT: 10.913 min **Area %: 100.0000 %**

HPLC TED-674

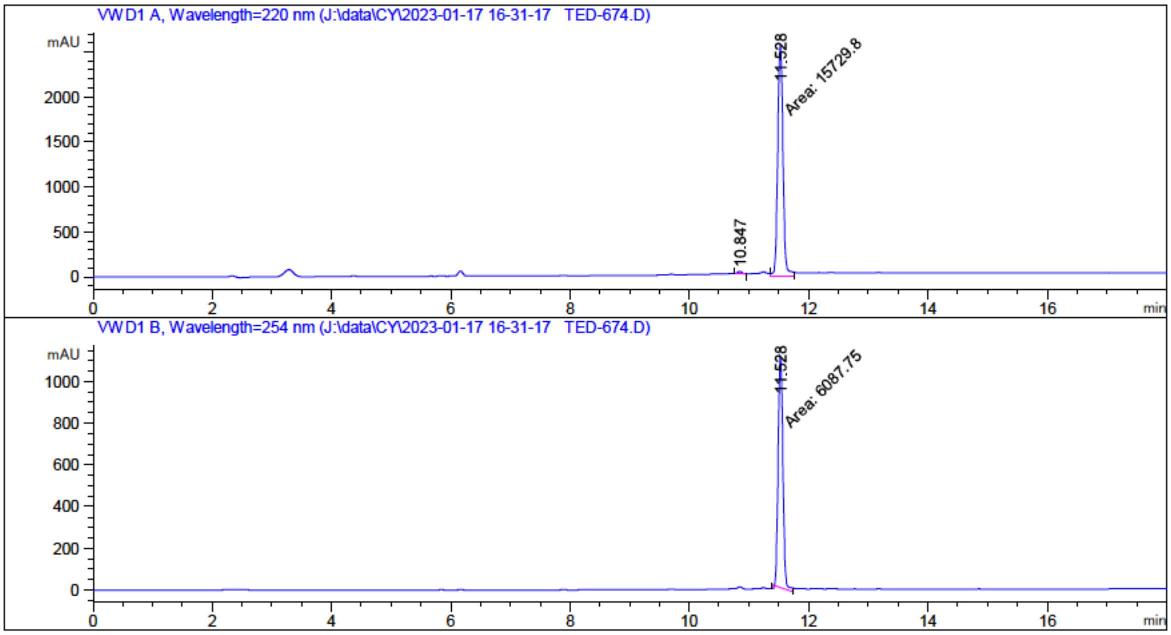

UV 220 nm:

RT: 10.847 min **Area %: 0.9279 %**

RT: 11.528 min **Area %: 99.0721 %**

UV 254 nm:

RT: 11.528 min **Area %: 100.0000 %**

HPLC TED-688

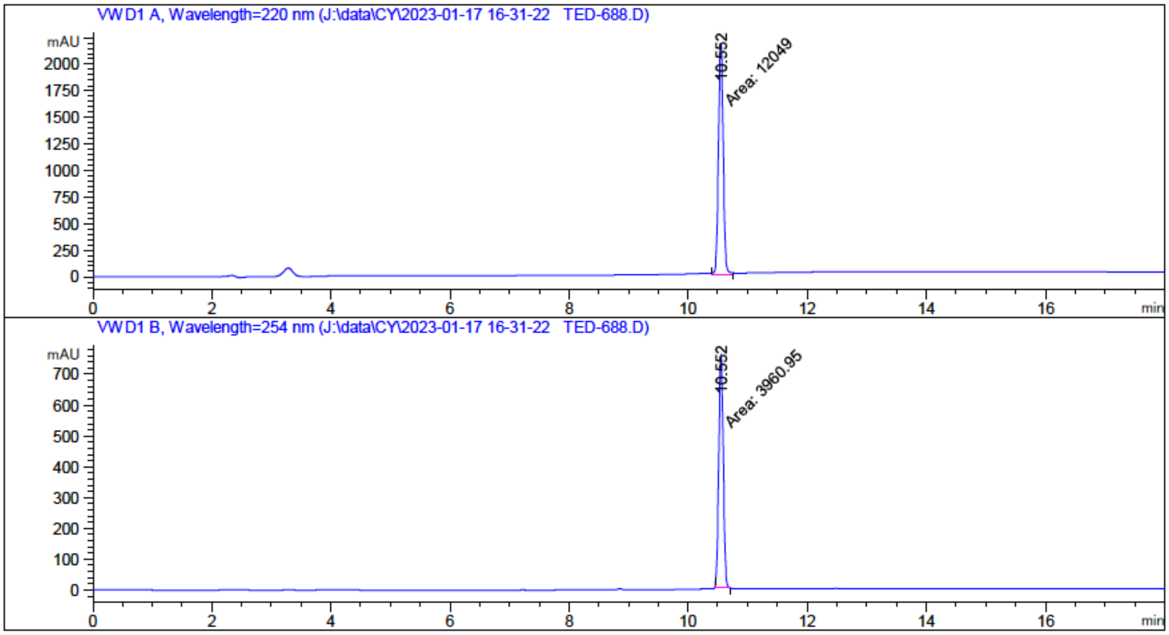

UV 220 nm:

RT: 10.552 min Area %: 100.0000 %

UV 254 nm:

RT: 10.552 min Area %: 100.0000 %

HPLC TED-689

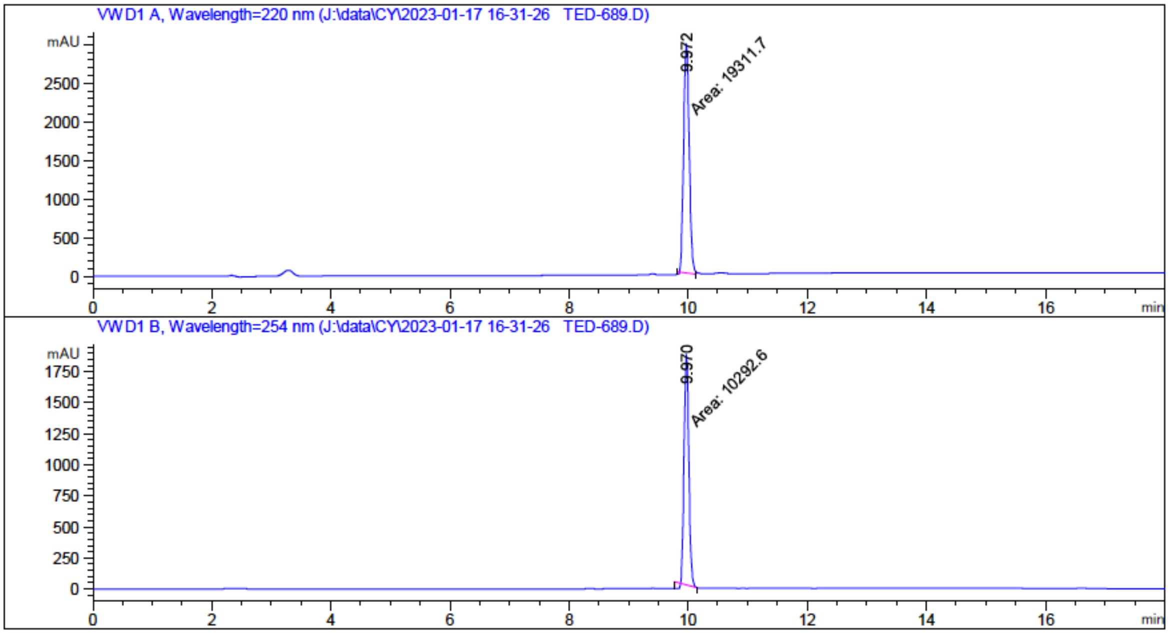

UV 220 nm:

RT: 9.972 min **Area %: 100.0000 %**

UV 254 nm:

RT: 9.970 min **Area %: 100.0000 %**

HPLC TED-690

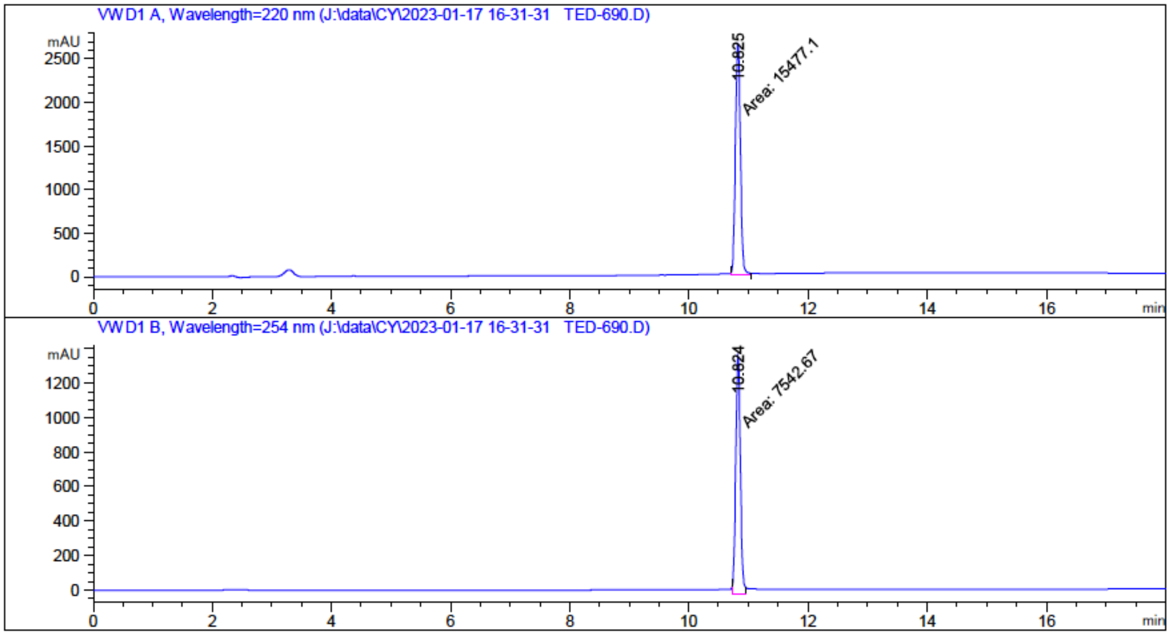

UV 220 nm:

RT: 10.825 min Area %: 100.0000 %

UV 254 nm:

RT: 10.824 min Area %: 100.0000 %

HPLC TED-734

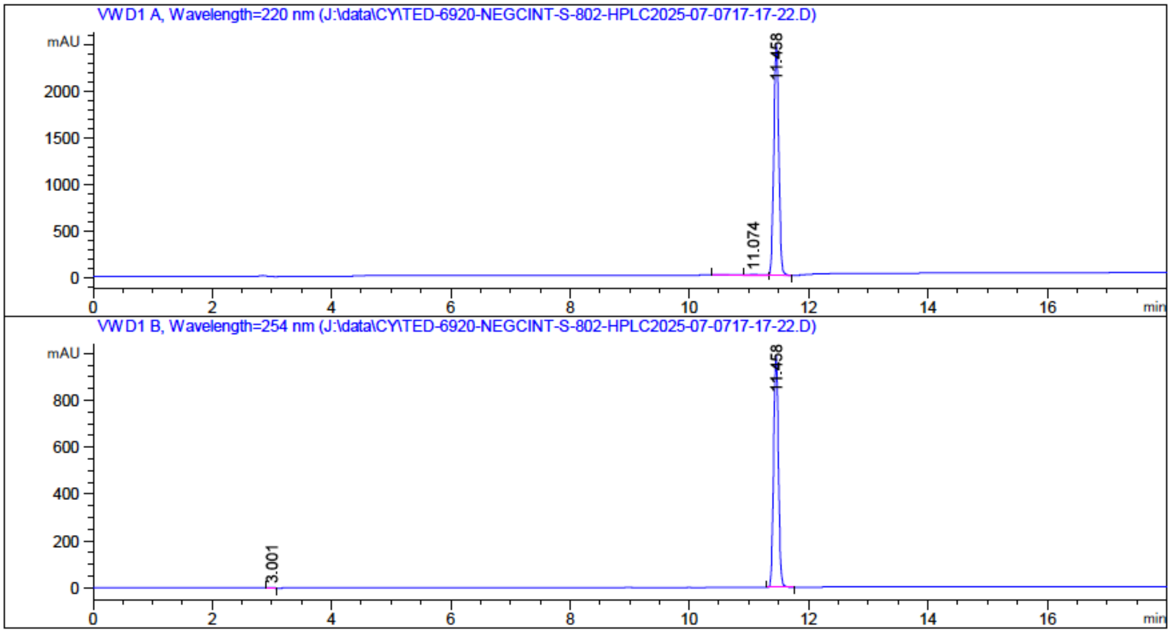

UV 220 nm:

RT: 11.074 min Area %: 0.9026 %

RT: 11.458 min Area %: 82.1328 %

RT: 18.771 min Area %: 16.9646 %

UV 254 nm:

RT: 3.001 min Area %: 0.1760 %

RT: 11.458 min Area %: 96.5651 %

RT: 18.769 min Area %: 3.2589 %
